# Comparative genomics of geographically distant *Fusarium fujikuroi* isolates revealed two distinct pathotypes correlating with secondary metabolite profiles

**DOI:** 10.1371/journal.ppat.1006670

**Published:** 2017-10-26

**Authors:** Eva-Maria Niehaus, Hee-Kyoung Kim, Martin Münsterkötter, Slavica Janevska, Birgit Arndt, Svetlana A. Kalinina, Petra M. Houterman, Il-Pyung Ahn, Ilaria Alberti, Stefano Tonti, Da-Woon Kim, Christian M. K. Sieber, Hans-Ulrich Humpf, Sung-Hwan Yun, Ulrich Güldener, Bettina Tudzynski

**Affiliations:** 1 Institute of Biology and Biotechnology of Plants, Molecular Biology and Biotechnology of Fungi, Westfälische Wilhelms-Universität Münster, Münster, Germany; 2 Department of Medical Biotechnology, Soonchunhyang University, Asan, Republic of Korea; 3 Institute of Bioinformatics and Systems Biology, Helmholtz Zentrum München, German Research Center for Environmental Health (GmbH), Neuherberg, Germany; 4 Institute of Food Chemistry, Westfälische Wilhelms-Universität Münster, Corrensstraße 45, Münster, Germany; 5 University of Amsterdam, Swammerdam Institute for Life Sciences, Plant Pathology, Amsterdam, The Netherlands; 6 Department of Agricultural Biotechnology, National Institute of Agricultural Sciences, Rural Development Administration, Wanju, Republic of Korea; 7 CREA-CIN Sede di Rovigo, Viale Giovanni Amendola, 82, 45100 Rovigo, Italy; 8 CREA-SCS Sede di Bologna, Via di Corticella, 133, 40128 Bologna, Italy; 9 Department of Energy Joint Genome Institute, University of California, Walnut Creek, Berkeley, California; 10 Chair of Genome-oriented Bioinformatics, TUM School of Life Sciences Weihenstephan, Technical University of Munich, Freising, Germany; Wageningen University, NETHERLANDS

## Abstract

*Fusarium fujikuroi* causes *bakanae* (“foolish seedling”) disease of rice which is characterized by hyper-elongation of seedlings resulting from production of gibberellic acids (GAs) by the fungus. This plant pathogen is also known for production of harmful mycotoxins, such as fusarins, fusaric acid, apicidin F and beauvericin. Recently, we generated the first *de novo* genome sequence of *F*. *fujikuroi* strain IMI 58289 combined with extensive transcriptional, epigenetic, proteomic and chemical product analyses. GA production was shown to provide a selective advantage during infection of the preferred host plant rice. Here, we provide genome sequences of eight additional *F*. *fujikuroi* isolates from distant geographic regions. The isolates differ in the size of chromosomes, most likely due to variability of subtelomeric regions, the type of asexual spores (microconidia and/or macroconidia), and the number and expression of secondary metabolite gene clusters. Whilst most of the isolates caused the typical *bakanae* symptoms, one isolate, B14, caused stunting and early withering of infected seedlings. In contrast to the other isolates, B14 produced no GAs but high amounts of fumonisins during infection on rice. Furthermore, it differed from the other isolates by the presence of three additional polyketide synthase (PKS) genes (*PKS40*, *PKS43*, *PKS51*) and the absence of the *F*. *fujikuroi*-specific apicidin F (NRPS31) gene cluster. Analysis of additional field isolates confirmed the strong correlation between the pathotype (*bakanae* or stunting/withering), and the ability to produce either GAs or fumonisins. Deletion of the fumonisin and fusaric acid-specific PKS genes in B14 reduced the stunting/withering symptoms, whereas deletion of the *PKS51* gene resulted in elevated symptom development. Phylogenetic analyses revealed two subclades of *F*. *fujikuroi* strains according to their pathotype and secondary metabolite profiles.

## Author summary

*Fusarium fujikuroi* causes *bakanae* disease of rice. Infected seedlings appear to be taller and more slender when compared to healthy seedlings due to its ability to produce gibberellic acids (GAs). The disease is responsible for high yield losses, and its incidence varies with regions, rice cultivars grown and the aggressiveness of the fungal isolates. However, not all infected seedlings show *bakanae* symptoms: one of the isolates, B14, causes stunting and early withering of infected seedlings. The reason for the two pathotypes is not well understood. Researchers thought that the stunting phenotype was mostly caused by fungal-derived secondary metabolites such as fusaric acid, but there is no experimental evidence yet. B14 differs from the other strains by the presence of more PKS gene clusters, low expression of GA genes, lack of detectable levels of GAs and the production of high amounts of fumonisins in rice. Analysis of additional field isolates revealed a strong correlation between the pathotype (*bakanae* or stunting) and either GA or fumonisin production. Based on phylogenetic analyses, *F*. *fujikuroi* strains can be divided into two phylogenetically distinct subclades according to their pathotype and secondary metabolite profiles. This study provides new insights into the genomic variations and the population structure inside the species *F*. *fujikuroi* which will help to develop disease control strategies for this rice pathogen.

## Introduction

The heterothallic ascomycete *Fusarium fujikuroi* Nirenberg is a member of the *Fusarium fujikuroi* species complex (FFC), a monophyletic lineage which includes at least eleven mating populations (MPs A-K) that are sexually infertile with one another, and numerous distinct anamorphic species [1].

*F*. *fujikuroi* (MP-C) is the causal agent of the rice disease *bakanae* (“foolish seedlings”), one of the most notorious seed-borne diseases with increasing economic importance in the major rice-growing countries in the world, including all rice-growing Asian and African countries, California, and more recently, Italy [1–3]. The fungus was one of the first fungal pathogens to be described, and *bakanae* is one of the oldest known diseases of rice being reported more than 100 years ago by Japanese scientists [4,5]. The most prominent symptoms of the disease are chlorotic, elongated and thin seedlings that are often several inches taller than healthy plants, and empty panicles leading to yield losses ranging from ca. 30–95% [6–9]. However, not all infected seedlings show the *bakanae* symptoms: sometimes they may be stunted or appear symptomless [10]. The incidence and severity of the *bakanae* or stunting disease symptoms varies with regions and isolate. The pathogen is dispersed predominantly with infected seeds, infected crop residues from the previous season in the soil, or by conidia on diseased stems which can be transmitted by rain and wind [6]. Disease control has become increasingly difficult due to rapidly developing fungicide resistance in the fungal population [4].

The enormous elongation of infected plants is caused by the ability of the pathogen to produce gibberellic acids (GAs), a family of plant hormones [11]. Fungal GAs are structurally identical to those synthesized by higher plants, but the respective biosynthetic pathways, genes and enzymes differ [12–14]. Previously we have shown that the ability of the fungus to produce GAs contributes to the efficient colonization in the rice roots [15]. In contrast to typical *bakanae* symptoms, it is unknown how the stunting phenotype of infected rice seedlings is triggered by *F*. *fujikuroi*. In addition, stunting of infected rice plants can also be caused by other *Fusarium* species, such as *F*. *proliferatum*, which can also be isolated from rice, though less abundantly than *F*. *fujikuroi* [16].

Recently, the first high-quality draft genome sequence of *F*. *fujikuroi* IMI 58289 has been published and the genetic capacity for biosynthesis of a whole arsenal of natural compounds has been demonstrated [15]. The genomes of additional *F*. *fujikuroi* isolates revealed some diversity regarding genome composition and virulence [17,18].

Here we present the genome sequences of eight additional *F*. *fujikuroi* strains, all but one isolated from infected rice from different geographic regions. The isolate FSU48 was obtained from maize. We provide a comparative analysis of the genome features, chromosome polymorphism, the ability to produce micro- and macroconidia, virulence, metabolome and transcriptome analyses under *in vitro* and *in planta* conditions. By the use of high-performance liquid chromatography coupled to a Fourier transform mass spectrometer (HPLC-FTMS) and genome-wide RNA-sequencing (RNA-seq), we demonstrate that in addition to species-specific common features there are differences between the isolates in all these aspects. Most importantly, we describe two pathotypes of *F*. *fujikuroi* on rice at the genomic, transcriptomic, and phylogenetic levels. Whereas most of the *F*. *fujikuroi* isolates caused typical *bakanae* symptoms with elongated chlorotic internodes, the isolate B14 caused stunting and withering of rice seedlings. We show that variations in the production of secondary metabolites (SMs), such as GAs and fumonisins, are crucial factors for the development of the *bakanae* or stunting pathotype, and that these two pathotypes are phylogenetically distinct groups among the field population of *F*. *fujikuroi*.

## Results and discussion

### Genome structure, gene families and the potential to produce secondary metabolites—common and different features

**Fig 1:**
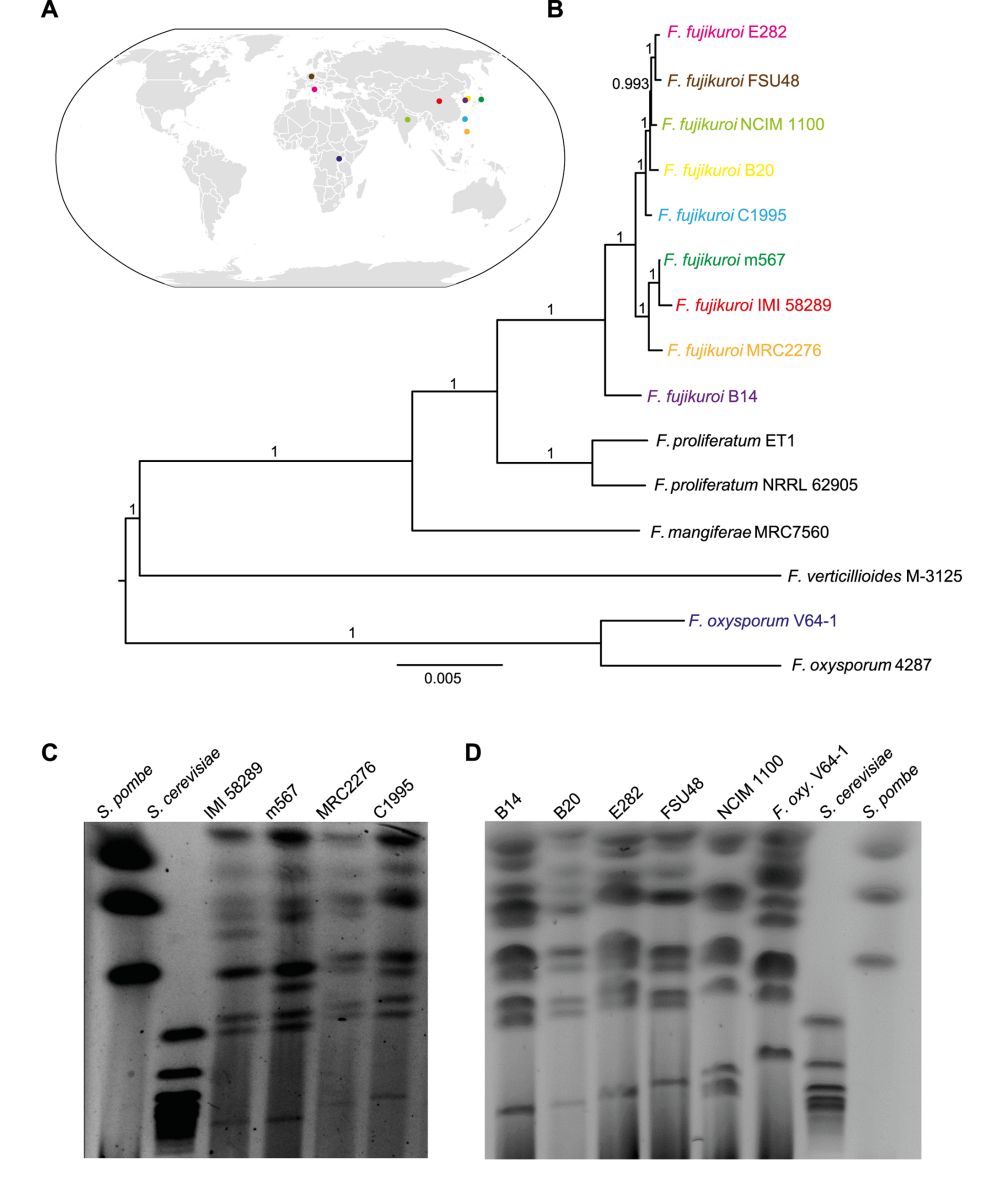
Phylogenetic relationships and chromosome characteristics of *F*. *fujikuroi* isolates and related species. (A) World map modified after https://commons.wikimedia.org/wiki/File:BlankMap-World6.svg. Colored dots present the origin of the different *F*. *fujikuroi* isolates (see Table 1). (B) Maximum likelihood tree showing phylogenetic relationships of *F*. *fujikuroi* isolates and other species representing the Asian, African and American clades of the *Fusarium fujikuroi* complex (FFC), as well as *F*. *oxysporum*, and the distantly related species *F*. *langsethiae* and *F*. *avenaceum*. It was calculated based on the protein sequences of 5,181 single copy genes that are shared among all analyzed species. (C) and (D) Separation of chromosomes of the nine *F*. *fujikuroi* isolates and *F*. *oxysporum* V-64-1 as outgroup on a CHEF gel. Chromosomes of *Schizzosaccharomyces pombe* and *Saccharomyces cerevisiae* were used as size standards.

*F*. *fujikuroi* is broadly distributed world-wide in all rice-growing countries. To gain insight to the level of variation regarding genome structure, morphology, asexual spore formation, virulence, expression profiles and secondary metabolism under laboratory conditions as well as on rice, we chose nine isolates from different areas of the world for comparative analysis of all these parameters (Table 1, Fig 1A).

**Table 1:** Strains sequenced in this study or used for comparison.

Strain	Species	Origin	Locus tag	ENA accessions	Reference
**Strains sequenced/analyzed in this study**					
IMI 58289	*F*. *fujikuroi*	China, infected rice	FFUJ	HF679023-HF679034	[15]
m567	*F*. *fujikuroi*	Japan, infected rice	FFM5	FMJV01000001-FMJV01000241	this study
MRC2276	*F*. *fujikuroi*	Philippines, infected rice	FFMR	FMJW01000001-FMJW01000028	this study
C1995	*F*. *fujikuroi*	Taiwan, infected rice	FFC1	FMJU01000001-FMJU01000086	this study
B14	*F*. *fujikuroi*	South Korea, infected rice	FFB14	FMSL01000001-FMSL01000066	[52,18]; this study
B20	*F*. *fujikuroi*	South Korea, infected rice	FFB20	FMJS01000001-FMJS01000318	[52]; this study
E282	*F*. *fujikuroi*	Italy, infected rice	FFE2	FMJT01000001-FMJT01000227	this study
FSU48	*F*. *fujikuroi*	Germany, maize	FFFS	FMSM01000001-FMSM01000182	this study
NCIM 1100	*F*. *fujikuroi*	India, infected rice	FFNC	FMJX01000001-FMJX01000240	this study
V64-1	*F*. *oxysporum*	Ruanda, infected rice	FRV6	FMJY01000001-FMJY01000049	this study
**Strains with publically available genome sequences used for comparison**					
KSU3368	*F*. *fujikuroi*	Thailand, infected rice	LW94		[17]
KSUX-10626	*F*. *fujikuroi*	USA, prairie grass	LW93		[17]
FGSC8932	*F*. *fujikuroi*	Taiwan, infected rice	Y057		[17]

The high quality genome sequence of strain IMI 58289 that was assembled into twelve scaffolds corresponding to the twelve chromosomes [15] was used as master genome for structural annotation and for comparative analysis. The rice isolate V64-1 from Ruanda appeared to be a *F*. *oxysporum* strain when the genome was sequenced and analyzed. Therefore, this strain was used as outgroup in this study. A phylogenetic tree including all so far sequenced *F*. *fujikuroi* isolates and other FFC members, as well as more distantly related *Fusarium* species is shown in Fig 1B. The tree was generated based on the protein sequences of 5,181 single copy genes by the fast approximate likelihood ratio test to calculate branch support (aLRT) [19], which is a fast and accurate alternative to the time-consuming bootstrap analysis. Strain KSUX10626 [17] seems to be outside the *F*. *fujikuroi* clade.

Although all newly sequenced strains except for V64-1 clearly belong to the species *F*. *fujikuroi*, visualization of chromosome content of the ten strains by pulse field gel electrophoresis (PFGE) combined with clamped homogeneous electric fields (CHEF) indicated that all strains contained multiple chromosomes of varying sizes (Fig 1C). However, the precise number of chromosomes in each strain could not be determined because several chromosomes had a similar size and could not be distinguished.

Table 2 summarizes physical genome features of the newly and previously (IMI 58289) sequenced strains. The genome size for the *F*. *fujikuroi* strains is in the range of 43.9 Mb (IMI 58289) to 46.1 Mb (E282 and FSU48). The outgroup genome *F*. *oxysporum* V64-1 is smaller (49.1 Mb) than the reference *F*. *oxysporum* strain 4287 (61.4 Mb). Most of these differences are likely due to a different read coverage and different completeness of assemblies for repetitive regions. Despite the varying number of protein-encoding genes (14,817 for IMI 58289 to 16,088 for E282) which partially may be due to a manual gene structure validation, most key genome features of the newly sequenced strains are similar to those of *F*. *fujikuroi* IMI 58289 [15].

**Table 2:** Comparative genome statistics for all sequenced *F*. *fujikuroi* isolates and *F*. *oxysporum* V64-1 (outgroup).

Strain	IMI 58289	m567	MRC 2276	C1995	B14	B20	E282	FSU48	NCIM 1100	V64-1
Locus tag	FFUJ	FFM5	FFMR	FFC1	FFB14	FFB20	FFE2	FFFS	FFNC	FRV6
Genome size (Mb)	43.9	44.0	45.0	45.8	44.0	44.3	46.1	46.1	45.3	49.1
Coverage	19	50	146	66	64	21	38	53	68	46
Scaffolds/Contigs	12	241	28	86	66	318	227	182	240	49
N50 scaffold (Mb)	4.2	1.9	4.5	4.2	3.0	0.27	2.1	2.1	2.3	5.2
GC-content (%)	47.4	47.1	47.5	47.0	48.1	48.2	47.0	46.9	46.9	47.1
Protein coding genes	14,817	15,371	15,904	15,920	15,852	15,940	16,088	16,054	15,725	16,976
Gene density (Number of genes per Mb)	337	349	353	348	360	360	349	348	347	346
Total exon length (Mb)	21.5	21.5	22.3	22.1	22.3	22.3	22.3	22.3	22.0	23.4
Total intron length (Mb)	1.9	2.0	2.3	2.3	2.0	2.0	2.1	2.1	2.1	2.6
Average distance between genes (kb)	1.4	1.3	1.3	1.3	1.2	1.3	1.3	1.3	1.4	1.4
Percent coding (%)	49.2	48.9	49.6	48.2	50.6	50.2	48.4	48.4	47.8	47.6
GC-content coding (%)	51.5	51.5	51.5	51.5	51.5	51.5	51.5	51.5	51.5	51.4
Average coding region length (kb)	1.5	1.4	1.4	1.4	1.4	1.4	1.4	1.4	1.4	1.4
Mean protein length (amino acids)	485	467	467	462	468	466	463	463	460	459
Exons	41,583	42,102	43,625	43,524	43,541	43,602	43,925	43,975	42,650	46,490
Average exon length (bp)	518	511	511	507	511	511	509	507	508	503
Exons/gene	2.81	2.7	2.7	2.7	2.7	2.7	2.7	2.7	2.7	2.7
Introns	26,769	26,730	27,721	27,604	27,689	27,662	27,837	27,921	26,923	29,514
Average intron length (bp)	69.2	75.5	83.7	81.7	71.7	70.6	74.4	76.8	74.6	87.0

The completeness of the new draft genomes was explored by comparing each predicted proteome to two different, highly conserved eukaryote protein sets by BLAST [20,21]. Orthologs for all conserved proteins were found in all proteomes with the exception of a missing ortholog to ‘T-complex protein 1 subunit theta’ and an ortholog to ‘translation factor eIF6’ in *F*. *fujikuroi* B20. In *F*. *oxysporum* V64-1, an ortholog to ‘MTO1 –mitochondrial translation optimization’ is missing suggesting that also these genomes are more than 99% complete. In addition, the protein sets were subjected to BUSCO analysis and subsequently compared to published *Fusarium* proteome sets [22]. Of the 3,725 single-copy orthologs searched (library Sordariomyceta_odb9), 97.9%–99.2% were detected as complete and single-copy in the *F*. *fujikuroi* strains and *F*. *oxysporum* V64-1 which even exceeds the completeness of *F*. *graminearum* PH1 (94.7%) and *F*. *oxysporum* (94.2%) (S1 Table).

Species of the FFC are heterothallic [23]. To determine the mating type of the nine *F*. *fujikuroi* isolates, we searched the genomes for the presence of genes either of the MAT1-1 or MAT1-2 mating types. Five of the strains contain the genes MAT1-1-1, MAT1-1-2 and MAT1-1-3, all belonging to the MAT1-1 mating type, while the other four contain the MAT1-2 mating type locus with the HMG-box type transcription factor (TF)-encoding gene *MAT1-2-1* and an additional gene, *MAT1-2-3*, that has been recently identified as specific to the Hypocreales [23] (S2 Table).

Differences between the isolates were found regarding the size of gene families prevalent in the genome of B14, which is the only one of the ten examined strains causing stunting of rice seedlings (see below). The number of transporter-encoding genes (954), Zn(II)_2_Cys_6_ fungal type TFs (567), cytochrome P450- (173) and dehydrogenase-encoding genes (404) were higher when compared to the other *F*. *fujikuroi* strains. Also the number of polyketide synthase (PKS) genes (18 plus two truncated pseudogenes) was larger than in the genomes of the other strains (Table 3).

**Table 3:** Distribution of selected gene families in genome sequences of *Fusarium fujikuroi* isolates and *F*. *oxysporum* V64-1 (outgroup).

Strain	IMI 58289	m567	MRC2276	C1995	B14	B20	E282	FSU48	NCIM 1100	V64-1
**Secondary metabolite** **biosynthetic genes**^**a**^										
PKS	14 (0|0)	14 (0|0)	15 (1|0)	14 (2|0)	18 (2|2)	15 (1|0)	14 (1|0)	13 (0|0)	9 (1|0)	10 (0|0)
PKS/NRPS	4 (0|0)	4 (0|0)	4 (0|0)	5 (0|0)	4 (0|0)	5 (0|0)	5 (0|0)	5 (0|0)	5 (0|0)	3 (0|0)
NRPS	16 (0|0)	16(0|0)	17 (0|0)	16 (1|0)	16 (1|0)	17 (1|0)	17 (1|0)	16 (0|0)	15 (0|0)	15 (0|0)
DMATS	2 (0|0)	2 (0|0)	3 (0|0)	3 (0|0)	3 (0|0)	3 (0|0)	3 (0|0)	3 (0|0)	3 (0|0)	3 (0|0)
TC	12 (3|0)	12 (2|0)	12 (1|0)	11 (0|0)	11 (1|0)	12 (1|0)	12 (1|0)	12 (1|0)	9 (1|0)	12 (0|0)
**Transporter genes**	879	880	931	916	954	924	930	919	886	1012
**Transcription factors (TFs)**	1,124	1,206	1,205	1,191	1,200	1,195	1,199	1,191	1,168	1,236
Zn(II)_2_Cys_6_ fungal type TFs (largest group of TFs)	511	544	555	548	567	547	550	540	521	551
**Other gene families**										
Histone modifying enzymes	119	125	122	121	122	127	123	124	127	130
Cytochrome P450	143	144	166	160	173	165	165	165	152	155
Monooxygenases	69	69	76	76	82	79	77	79	69	82
Oxidoreductases	47	46	43	46	48	47	47	47	45	42
Protein kinases and phosphatases	250	260	266	264	273	268	268	268	276	268
Dehydrogenases	370	370	392	388	404	393	395	396	369	469
Glycoside hydrolases	314	315	331	323	338	329	329	330	310	376

^**a**^ Secondary metabolite gene predictions are based on InterPro domains, manually validated and corrected based on reports in the literature and on comparative analysis of fusaria. Values are the number of genes per genome; values in brackets are number of pseudogenes/remnants; | genes unique to a genome among the strains examined. PKS—polyketide synthase, NRPS—non-ribosomal peptide synthetase, DMATS—dimethylallyltryptophan synthase, TC–terpene cyclase

The strain NCIM 1100 encodes less PKS (eight plus one truncated pseudogene) and nine terpene cyclase (TC) genes which results in the inability to produce gibepyrones, fujikurin and certain terpenes besides other unknown products (S3 Table and below).

To gain a deeper insight into the variation at the chromosome ends between the ten analyzed strains, we used a PCR approach with primer pairs from the two ends of each chromosome based on the genome sequence of strain IMI 58289 [15]. This analysis revealed differences between the isolates either on one side or on both sides of the chromosomes (S1 Fig). The most variations were found for subtelomeric regions of chromosomes 3, 5, 7, 10 and 11. Two of the key enzyme-encoding genes of SM gene clusters, NRPS31 (apicidin F) and PKS16 (unknown product), are located at the far end of chromosome 1 and 11, respectively, and the PCR analysis clearly showed differences in the presence of these SM genes (S1 Fig).

### Variation in growth, colony morphology and asexual spore formation

**Fig 2:**
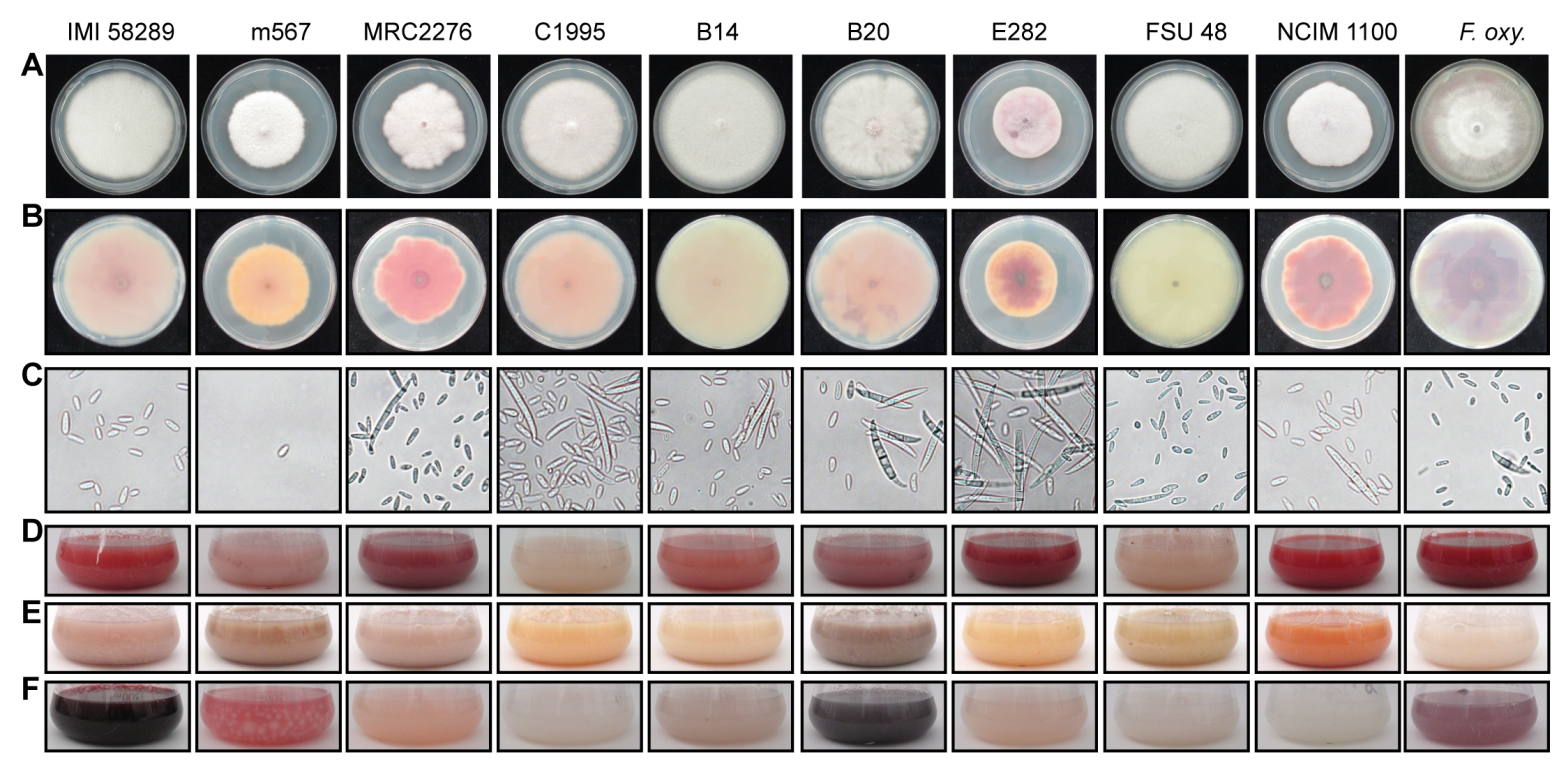
Phenotypic characteristics of the nine *F*. *fujikuroi* isolates and *F*. *oxysporum* V64-1 as outgroup. Colony morphology of the strains grown on solidified complete medium from the top (A) and the bottom (B) of the plates. Variation in pigmentation of the strains grown in liquid synthetic medium containing 6 mM glutamine (C) (optimal for bikaverin), 60 mM glutamine (D) or 6 mM NaNO_3_ (E) (optimal for fusarubins). (F) Microscopic images of microconidia and macroconidia.

All isolates showed differences in colony morphology and pigmentation on solidified and in liquid media, respectively, indicating metabolic variation between them (Fig 2A–2E). There were differences in the ability to produce the red pigments, bikaverin and fusarubins, despite the presence of the respective gene clusters in all *F*. *fujikuroi* isolates (Fig 2C–2E). Previously, we have shown that both PKS-derived pigments are only produced under low nitrogen conditions. However, while bikaverin biosynthesis is induced at acidic pH, the perithecial pigments fusarubins are produced only under alkaline pH conditions [24,25]. Strain C1995 showed no coloration under bikaverin-production conditions (Fig 2C), and C1995, B14, FSU48 and NCIM 1100 showed no pigmentation under fusarubin production conditions (Fig 2E). To verify that the biosynthetic genes are not expressed in these isolates, we performed Northern blot analyses using the bikaverin and fusarubin biosynthetic genes as probes (S2 Fig). *FSR2* (encoding an *O*-methyltransferase in the fusarubin gene cluster) was expressed in IMI 58289, but not in the remaining strains suggesting that the fusarubin genes are only slightly expressed in m567, MRC2276, B20, E282 and *F*. *oxysporum* V64-1, or alternatively that the red pigmentation in these strains under fusarubin production conditions (Fig 2F) might be due to the expression of the bikaverin biosynthetic genes.

The species *F*. *fujikuroi* is described to produce slender macroconidia with three to five septa and oval or club-shaped microconidia, mostly without or with one septum [1]. However, the strains used in this work differ in their ability to produce asexual spores, i.e. micro- and macroconidia (Fig 2F). While most of the strains produce both types of conidia, others produce predominantly (B14 and NCIM 1100) or exclusively (IMI 58289) microconidia or predominantly macroconidia (E282), respectively, on V8 agar under light conditions. Strain m567 hardly forms any spores. In *A*. *nidulans*, asexual reproduction has been extensively studied for several decades [26–28]. Sequential activation of three major regulators, BrlA, AbaA and WetA, is necessary for the fungus to undergo asexual development. In addition, there are a number of *fluffy* regulatory genes (*FLBA–FLBE*) which regulate *BRLA* expression. Recently, it has been shown that an AbaA-WetA pathway is conserved in the distantly related species *Fusarium graminearum* [29–31]. There are highly conserved homologs for FlbB, FlbC, FlbD, FlbE and the central regulators WetA and AbaA also in the genomes of the *F*. *fujikuroi* isolates. However, no close relative to BrlA, which is responsible for the vesicle formation during conidiogenesis in *A*. *nidulans*, has been found in any *Fusarium* genome.

### The potential to produce a broad spectrum of secondary metabolites

In recent years, several new SMs have been identified in *F*. *fujikuroi* due to the deciphering of the fungal genome and the application of molecular techniques to activate silent gene clusters, e.g. those for apicidin F, fujikurins, beauvericin, trichosetin, and reversely *N*-prenylated tryptophan (r-*N*-DMAT) [32–36] as well as for the sesquiterpenes eremophilene and guaia-6,10(14)-diene [37,38]. In addition, well known SMs such as fusaric acid, fusarins, fusarubins and gibepyrones have been linked to the respective biosynthetic gene clusters [39–42].

**Fig 3:**
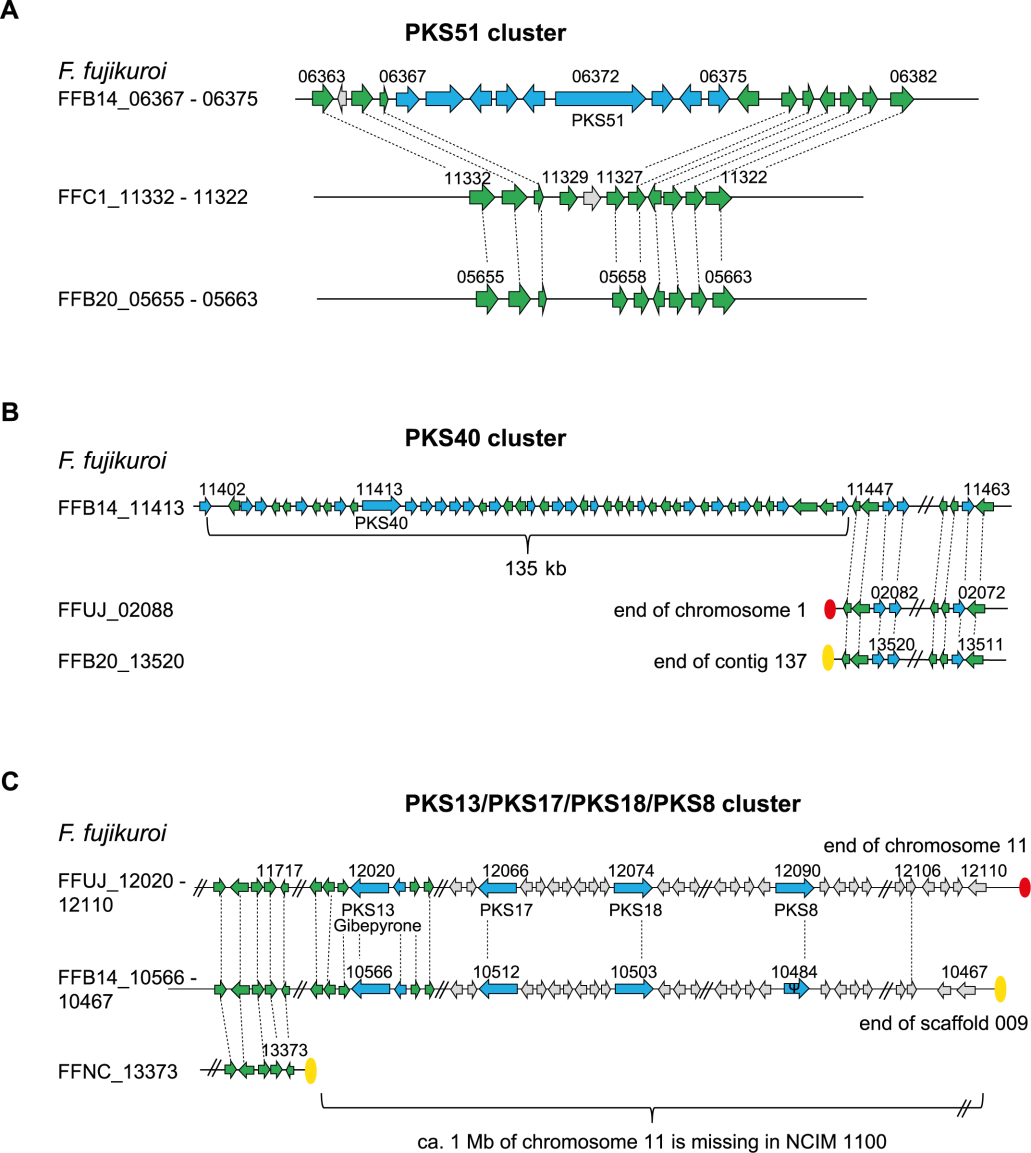
Content and arrangement of genes in different gene clusters with variations in the single isolates. The PKS51 (A) and PKS40 (B) gene clusters are present only in strain B14. (C) The PKS13, PKS17, PKS18, and PKS8 gene clusters at the end of chromosome 11 are missing in strain NCIM 1100. Arrows in blue represent genes belonging to a specific gene cluster. Green arrows represent genes that have closely related homologs in two or more isolates while light-gray arrows represent genes that do not have closely related homologs in other isolates. Ψ indicates a pseudogene.

**Fig 4:**
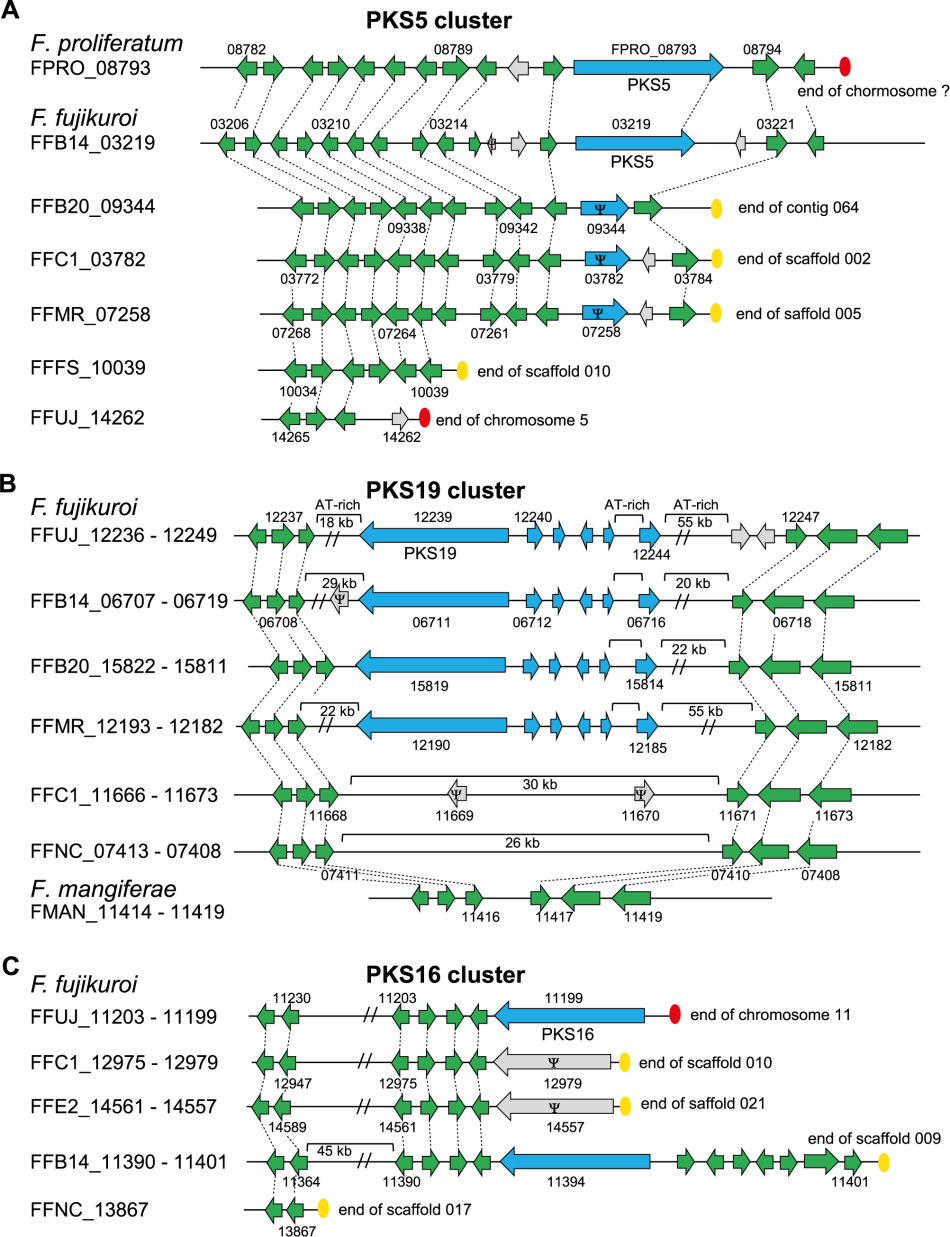
Content and arrangement of genes in different gene clusters with variations in the single isolates. (A) The PKS5 cluster seems to be functional only in strain B14. The PKS5-encoding gene is truncated or missing in the other isolates. (B) The PKS19 (fujikurin) gene cluster is present in some and totally missing in other isolates. (C) *PKS16* at the end of chromosome 11 in *F*. *fujikuroi* IMI 58289 is truncated in stains C1995 and E282, and missing in strain NCIM 1100. Arrows in blue represent genes belonging to a specific gene cluster. Green arrows represent genes that have closely related homologs in two or more isolates while light-gray arrows represent genes that do not have closely related homologs in other isolates. Ψ indicates a pseudogene.

Bioinformatic analysis of the nine *F*. *fujikuroi* strains revealed several differences in the presence of PKS, NRPS, dimethylallyltryptophan synthase (DMATS) and TC gene clusters between them and also compared to closely related FFC members, such as *F*. *mangiferae* and *F*. *proliferatum* [43] (S3 Table). Altogether, there are 17 NRPS-, 23 PKS-, three DMATS-, and twelve TC-encoding genes present in the nine *F*. *fujikuroi* strains, i.e. 55 unique core-enzyme-encoding genes that could give rise to 54 distinct SMs (the fusaric acid cluster encodes two core enzymes, PKS6 and NRPS34). Only twelve of the 23 PKS-, 13 of the 18 NRPS-, eight of the twelve TC- and two of the three DMATS-encoding genes are present in the genomes of all *F*. *fujikuroi* isolates. The Korean strain B14 has three additional PKS genes (*PKS40*, *43* and *51*) not present in any other analyzed *F*. *fujikuroi* strain (S3 Table, Fig 3A and 3B). PKS51 is also not present in any other member of the FFC, while PKS40 is present in *F*. *proliferatum* ET1 and *F*. *verticillioides*, and PKS43 in *F*. *mangiferae*, respectively (S3 Table). In addition, B14 is the only *F*. *fujikuroi* isolate with a complete PKS5 cluster. This yet uncharacterized cluster is either absent or contains pseudogenes in the other isolates of this species, but seems to be functional in *F*. *proliferatum* and *F*. *mangiferae* (Fig 4A). The fujikurin gene cluster (PKS19) has been previously described as *F*. *fujikuroi*-specific [15], but was recently discovered in two newly sequenced strains of *F*. *proliferatum* [43]. However, the fujikurin cluster is absent in three of the nine analyzed *F*. *fujikuroi* isolates (S3 Table, Fig 4B). The apicidin F gene cluster (NRPS31) which is located at the far end of chromosome 1 in strain IMI 58289 [15] was shown to be unique for *F*. *fujikuroi* [32]. This cluster is present in all isolates but one, B14, most likely due to chromosome rearrangements in the subtelomere regions (S1 and S3A Figs). While the entire fumonisin gene cluster (PKS11) is present in most members of the FFC except for *F*. *mangiferae* (S3 Table), nine genes of the cluster, the homologs of *FFUJ_09248* –*FFUJ_09256*, are missing in *F*. *fujikuroi* B20 (S3B Fig) and C1995.

The Indian isolate NCIM 1100 has only 14 PKS-encoding genes (S3 Table). The absence of five PKS genes/clusters is due to large parts of chromosome 11 missing at both ends in this strain compared to IMI 58289 (Figs 3C and 4C, S1 Fig). The right border of chromosome 11 contains *PKS8* (*FFUJ_12090*), *PKS13* (*FFUJ_12020*), *PKS17* (*FFUJ_12066*), and *PKS18* (*FFUJ_12074*), while *PKS16* (*FFUJ_11199*) and adjacent genes are absent at the left border of this chromosome (Figs 3C and 4C, S3 Table). Also the discontinuous distribution of some other SM gene clusters among the *F*. *fujikuroi* isolates is likely the result of gene loss and gene gain events, respectively, at the chromosome ends. However, gene clusters located in central parts of chromosomes, e.g. PKS19 (fujikurin) and PKS11 (fumonisin) clusters, are also only present in some and absent in other strains, while the genes at the right and left borders of the clusters show colinearity between all isolates. It is not clear whether these gene clusters are the result of horizontal gene transfer [44] or cluster duplication and loss (birth and death) as shown for the fumonisin gene cluster [45].

Thanks to the genome sequencing and subsequent identification of putative gene clusters [15], SM products have now been assigned to 22 of the 54 predicted PKS-, NRPS-, DMATS-, and TC-derived SM gene clusters in isolates of *F*. *fujikuroi*, and an additional two (ferrirhodin and depudecin) in members of the FFC. One reason for the inability to identify more products is probably due to the fact that many of the SM genes are not or only minimally expressed under laboratory conditions [46]. Only 15 of these predicted clusters with known products are present in the genomes of all nine *F*. *fujikuroi* isolates indicating the genetic diversity among isolates of one species. The genes which are commonly present in all *F*. *fujikuroi* genomes are four NRPS genes required for synthesis of the siderophores ferricrocin and fusarinine and the mycotoxins beauvericin and fusaric acid (together with PKS6); six PKS genes required for synthesis of fusarubins, bikaverin, fusaric acid (together with NRPS8), fusarins, trichosetin, and fumonisins; six TCs required for synthesis of GAs, phytoene, eremophilene, (–)-α-acorenol, (–)-guaia-6,10(14)-diene, and (+)-koraiol, and one DMATS responsible for biosynthesis of r-*N*-DMAT (S3 Table).

To study the variability in the production of the most prominent SMs, we cultivated the strains under three standardized culture conditions (6 mM and 60 mM glutamine, 6 mM NaNO_3_). The optimal conditions for the production of the different metabolites were previously shown to vary considerably regarding nitrogen availability and pH in strain IMI 58289 [15]. Therefore, we analysed these SMs in the three media by high-performance liquid chromatography coupled to a Fourier transform mass spectrometer (HPLC-FTMS) or by HPLC with a diode array detector (HPLC-DAD) in the case of GAs. We compared the production levels (Table 4) with transcriptome profiles of the SM biosynthetic genes generated by RNA-seq for two of these conditions (6 mM and 60 mM glutamine) (Table 5A and 5B). For most of the SMs, we observed similar regulatory mechanisms regarding nitrogen availability as previously described for strain IMI 58289. However, there were strain-specific differences.

**Table 4:** Secondary metabolite production of the ten analyzed strains in submerged culture at three different nitrogen and pH conditions.

Strain	BIK	FSR	FSA	GPY	APF	BEA	FB_2_	FB_1_	FusC	FUJ	GA_3_	GA_4_	GA_7_
**6 mM glutamine**													
**IMI 58289**	+++	-	-	++	-	-	-	(+)	-	-	++	+	++
**m567**	+	-	-	+	-	-	-	-	-	-	+++	+	++
**MRC2276**	++	-	-	-	-	-	-	-	-	-	++	+	+
**C1995**	-	-	-	-	-	-	-	-	-	-	++	++	++
**B14**	+	(+)	-	-	n.p.	-	+++	+++	-	-	(+)	-	-
**B20**	++	+	-	-	-	(+)	-	-	-	-	++	+	++
**E282**	++	-	-	+	-	-	-	-	-	-	++	++	++
**FSU48**	-	-	(+)	-	-	-	-	-	(+)	-	++	++	+++
**NCIM 1100**	++	-	-	-	+	(+)	-	-	-	-	+++	+	++
***F*. *oxy*. V64-1**	+	-	-	+	-	-	-	-	-	-	-	-	-
**60 mM glutamine**													
**IMI 58289**	+	-	+++	++	+	-	-	-	+	-	(+)	-	(+)
**m567**	+	-	++	++	++	-	-	-	++	-	+	+	+
**MRC2276**	+	-	++	++	++	-	-	-	+	-	(+)	(+)	(+)
**C1995**	-	-	++	++	+	(+)	-	-	++	-	+	+	+
**B14**	(+)	-	+	-	n.p.	+	(+)	+	+	-	-	-	-
**B20**	++	(+)	+	(+)	+++	+++	-	(+)	++	(+)	+	+	+
**E282**	-	-	+	+++	+	-	-	-	++	-	+	(+)	-
**FSU48**	-	-	++	+	+	+	-	-	++	-	+	+	+
**NCIM 1100**	+	-	++	-	+	+	-	-	+++	-	+	+	+
***F*. *oxy*. V64-1**	-	-	++	++	-	-	-	-	-	-	(+)	-	-
**6 mM NaNO**_**3**_													
**IMI 58289**	++	+++	-	++	-	(+)	-	-	-	-	++	++	++
**m567**	-	+	+	-	-	-	-	-	-	-	+++	++	+++
**MRC2276**	-	(+)	-	-	-	-	-	-	-	-	+	++	++
**C1995**	-	-	++	-	-	-	-	-	-	-	++	+++	++
**B14**	-	+	+	-	n.p.	-	-	+	-	+++	-	-	-
**B20**	+	++	+	-	-	+	-	-	-	-	+	++	++
**E282**	(+)	-	+	-	-	-	-	-	-	-	++	++	++
**FSU48**	-	-	+	(+)	-	-	-	-	-	-	++	++	++
**NCIM 1100**	-	-	+	-	-	-	-	-	-	-	++	++	++
***F*. *oxy*. V64-1**	+	(+)	-	-	-	-	-	-	-	-	-	-	-

**+++**: 90–100% of the max. value; **++**: 10–90% of the max. value; **+**: <10%; **(+)**: not present in all triplicates; n.p.: cluster not present; BIK–bikaverin, FSR–*O*-methylfusarubin, FSA–fusaric acid, GPY–gibepyrone A, APF–apicidin F, BEA–beauvericin, FB1 and FB_2_ –fumonisins B1 and B_2_, FusC–fusarin C, FUJ–fujikurin A, GA_3/4/7_ –gibberellic acids GA_3/4/7_.

**Table 5:** Expression of the secondary metabolite key enzyme-encoding genes under high (A) and low (B) nitrogen conditions and in rice roots after 7 days post inoculation (dpi). n.p.–cluster not present.

A) High nitrogen (60 mM glutamine)										B) Low nitrogen (6 mM glutamine)										C) Rice (7 dpi)											
FF UJ	FF M5	FF MR	FF C1	FF B14	FF B20	FF E2	FF FS	FF NC	FR V6	FF UJ	FF M5	FF MR	FF C1	FF B14	FF B20	FF E2	FF FS	FF NC	FR V6	FF UJ	FF M5	FF MR	FF C1	FF B14	FF B20	FF E2	FF FS	FF NC	FR V6	Key enzyme	Product
2.0	0.8	1.5	1.4	2.3	0.9	0.6	2.4	1.1	3.4	1.6	0.7	1.5	0.8	2.3	1.7	0.9	0.6	1.8	1.3	3.0	2.2	2.8	2.6	3.6	2.9	1.9	3.1	3.1	2.5	DMATS1	r-*N*-DMAT
0.0	0.0	0.7	0.0	0.0	4.1	0.0	1.5	0.0	0.0	0.7	0.0	2.9	0.8	0.1	1.9	0.0	0.6	0.0	0.0	0.8	1.3	2.5	1.3	4.0	2.9	0.3	2.0	0.7	0.9	DMATS3	
n.p.	n.p.	0.8	1.4	0.7	2.0	0.4	1.5	1.3	0.5	n.p.	n.p.	0.3	4.1	0.6	0.9	0.1	2.0	0.2	0.0	n.p.	n.p.	1.4	2.5	1.2	1.7	0.9	1.5	0.9	1.6	DMATS4	
0.4	3.8	2.0	3.2	0.1	6.6	1.1	1.1	6.2	0.0	10.6	11.3	9.8	10.8	3.5	11.1	11.7	11.7	11.6	0.4	9.5	9.8	10.4	9.3	4.0	11.0	9.6	10.0	9.2	0.1	DTC1-1	Gibberellins
0.1	0.1	0.3	0.5	0.3	2.0	0.9	0.3	n.p.	0.0	0.3	0.1	3.3	3.3	2.5	6.9	0.4	1.2	n.p.	0.0	0.4	1.7	4.7	1.9	7.5	3.7	0.6	4.0	n.p.	0.3	iaaH	Auxins
0.1	0.0	0.0	0.1	0.1	1.3	0.0	0.0	n.p.	0.0	0.1	0.1	0.2	3.8	2.6	6.7	0.0	0.5	n.p.	0.0	0.4	1.5	5.1	0.9	7.5	4.0	0.6	4.0	n.p.	0.0	iaaM	Auxins
0.0	0.0	0.0	0.0	0.0	0.3	0.0	0.1	0.0	n.p.	0.0	0.0	0.0	0.1	0.0	0.0	0.0	0.1	0.0	n.p.	0.0	0.0	0.1	0.2	0.0	0.2	0.0	0.0	0.0	n.p.	IPT_LOG1	Cytokinins
0.0	0.3	0.0	0.0	0.0	0.4	0.1	0.0	0.3	0.0	0.0	0.1	0.1	0.1	0.2	0.0	0.0	0.0	0.2	0.0	0.1	0.1	0.5	0.2	1.3	1.3	0.0	0.4	0.3	0.8	IPT_LOG2	Cytokinins
n.p.	n.p.	0.0	n.p.	0.0	0.0	0.0	n.p.	0.0	0.0	n.p.	n.p.	0.0	n.p.	0.0	0.0	0.0	n.p.	0.0	0.0	n.p.	n.p.	0.0	n.p.	0.3	0.1	0.1	n.p.	0.4	0.1	NRPS01	Malonichrom
1.5	3.5	3.7	2.9	4.4	5.2	3.2	2.5	2.5	2.6	4.9	6.1	5.5	3.9	6.6	6.3	5.8	3.9	4.8	5.6	3.4	4.8	3.9	3.4	4.2	3.5	3.5	3.2	3.5	2.7	NRPS02	Ferricroin
1.1	1.3	1.3	1.6	2.0	2.0	1.3	0.5	1.0	2.3	3.3	3.3	2.8	2.1	3.2	3.0	3.3	1.9	3.1	3.9	2.0	2.1	2.4	2.1	1.9	2.2	2.1	1.9	2.1	2.2	NRPS03	
0.0	1.8	0.2	0.2	0.2	4.5	0.0	1.2	0.0	0.0	0.0	0.0	0.0	0.0	0.0	0.2	0.0	0.0	0.0	0.0	0.3	2.5	2.4	0.3	3.1	3.0	0.2	1.5	0.2	0.8	NRPS04	
6.9	6.8	6.5	7.7	7.3	7.3	7.5	5.9	7.0	5.0	3.0	3.8	3.1	4.6	3.0	3.3	6.8	4.3	7.5	2.1	4.4	4.9	4.1	4.2	3.7	2.8	6.3	4.1	4.8	4.2	NRPS06	Fusarinine
4.0	3.5	3.0	4.5	3.5	4.1	2.8	4.3	3.6	2.7	4.9	3.9	4.2	4.2	4.7	3.8	4.5	3.7	6.3	4.8	4.8	4.8	4.0	3.3	4.8	4.9	4.8	3.9	5.5	3.5	NRPS10	
0.1	0.0	0.1	0.4	0.1	1.7	0.4	0.1	0.3	0.2	4.4	5.6	0.8	5.1	1.5	0.9	0.7	0.6	4.2	1.4	3.1	1.9	3.3	4.6	2.7	1.9	3.3	3.2	3.3	4.1	NRPS11	
0.0	0.0	0.0	0.0	0.0	0.0	0.0	0.0	0.0	0.0	0.0	0.0	0.6	0.0	0.3	0.0	0.1	0.0	0.0	0.1	0.3	0.3	2.5	2.1	2.9	2.4	0.2	3.0	0.1	0.6	NRPS12	
1.4	1.3	6.3	2.9	6.8	3.9	2.9	2.6	4.4	5.6	1.1	2.1	3.0	1.6	2.6	3.6	6.3	1.3	3.9	3.8	1.1	1.3	3.6	1.8	3.5	3.1	1.7	2.2	2.7	1.2	NRPS13	
0.0	0.0	0.0	0.0	0.0	0.0	0.0	0.0	n.p.	n.p.	0.0	0.0	0.0	0.0	0.0	0.0	0.0	0.0	n.p.	n.p.	0.0	0.0	0.0	0.0	0.0	0.2	0.0	0.0	n.p.	n.p.	NRPS17	Ferrichrom
0.0	0.0	0.0	0.0	0.0	0.0	0.0	0.0	0.0	0.0	0.0	0.0	0.0	0.0	0.0	0.0	0.0	0.0	0.0	0.0	0.1	0.0	0.0	0.1	0.0	0.1	0.0	1.0	0.0	0.0	NRPS20	
0.0	0.0	0.2	0.1	0.0	0.1	0.0	0.2	0.0	0.0	0.0	0.0	0.0	0.0	0.1	0.0	0.0	0.0	0.0	0.0	0.2	0.1	0.0	0.0	0.0	0.0	0.1	0.1	0.1	0.2	NRPS21	
0.1	0.1	0.2	0.8	0.7	8.3	0.3	3.8	0.1	0.0	0.2	0.1	0.2	2.0	0.7	7.3	0.2	2.1	0.7	0.0	0.1	0.2	0.6	0.2	3.3	4.0	0.1	1.1	0.1	3.5	NRPS22	Beauvericin
0.4	0.3	0.7	1.1	0.5	3.0	0.4	1.4	n.p.	0.2	2.5	2.0	2.4	3.4	2.2	2.7	2.2	2.7	n.p.	2.0	0.3	0.3	0.7	0.9	0.7	1.2	0.2	0.2	n.p.	2.5	NRPS23	
0.1	0.1	0.0	0.0	0.4	0.0	0.1	0.0	0.3	n.p.	0.0	0.0	0.0	0.1	0.0	0.0	0.0	0.0	0.1	n.p.	0.0	0.0	0.0	0.0	0.0	0.0	0.0	0.0	0.0	n.p.	NRPS25	
3.9	4.4	5.4	5.6	n.p.	6.7	5.0	4.0	5.4	n.p.	0.0	0.0	0.0	0.1	n.p.	0.1	0.1	0.0	0.5	n.p.	2.7	3.9	1.7	0.5	n.p.	2.1	2.4	3.0	2.8	n.p.	NRPS31	Apicidin F
6.6	3.5	7.1	6.2	3.9	8.8	5.6	9.1	6.9	0.0	0.3	0.3	0.3	7.4	0.3	0.4	0.3	7.8	2.5	0.0	4.4	5.9	6.8	6.7	5.7	6.9	7.1	6.4	5.6	4.8	NRPS34	Fusaric acid
n.p.	n.p.	n.p.	0.0	n.p.	0.0	0.1	0.0	0.1	n.p.	n.p.	n.p.	n.p.	0.1	n.p.	0.5	0.0	0.0	0.0	n.p.	0.0	0.0	0.0	0.2	0.0	0.0	0.1	0.0	0.0	0.0	PKS/NRPS50	Fusaridone A
0.3	0.1	0.6	1.6	0.0	1.5	0.3	0.5	0.2	0.1	0.0	0.0	0.0	2.3	0.0	0.1	0.0	0.2	0.0	0.2	0.0	0.0	0.1	0.3	0.8	2.2	0.0	0.0	0.0	0.1	PKS01	Trichosetin
0.2	0.0	0.0	0.0	2.2	0.6	0.0	0.0	0.0	0.0	0.0	0.0	0.0	0.0	0.4	0.0	0.0	0.0	0.0	0.0	0.9	1.0	0.5	0.2	1.2	0.3	0.4	0.2	1.0	2.2	PKS02	
0.1	0.0	0.5	0.1	0.4	0.2	0.2	0.2	0.2	0.0	0.1	0.1	0.0	0.2	0.2	0.2	0.6	0.0	0.5	0.1	0.2	0.8	0.5	0.3	0.5	0.2	0.7	0.3	0.3	1.9	PKS03	Fusarubins
0.8	5.7	2.6	1.0	0.2	5.1	0.3	1.7	1.8	0.1	9.1	8.9	9.6	5.1	6.4	6.1	9.2	4.8	8.9	9.5	1.8	0.5	0.2	0.1	0.2	0.0	4.2	0.1	3.8	3.8	PKS04	Bikaverin
n.p.	n.p.	0.0	0.1	0.1	0.0	n.p.	n.p.	0.0	0.0	n.p.	n.p.	0.0	0.0	0.0	0.0	n.p.	n.p.	0.0	0.0	n.p.	n.p.	0.0	0.0	0.1	0.0	n.p.	n.p.	0.0	0.1	PKS05	
7.6	3.7	6.4	6.7	4.7	8.6	6.2	9.4	6.7	0.4	1.1	1.0	0.5	8.6	0.6	0.8	0.9	9.1	3.3	0.4	4.4	6.1	6.9	6.9	5.2	6.7	7.1	6.5	5.4	3.7	PKS06	Fusaric acid
0.1	0.1	0.1	0.3	0.2	0.1	0.2	0.0	0.2	4.6	1.6	1.2	0.6	0.1	2.0	0.8	1.3	0.2	0.6	1.7	0.1	0.2	0.3	0.2	0.1	0.1	0.3	0.2	0.2	2.5	PKS07	
0.0	0.0	0.0	0.0	0.0	0.0	0.0	0.0	n.p.	n.p.	4.2	1.2	5.6	3.6	6.2	0.1	6.3	4.1	n.p.	n.p.	0.1	0.1	0.1	0.1	0.2	0.0	0.1	0.1	n.p.	n.p.	PKS08	
3.2	0.2	1.0	3.3	2.2	0.7	0.7	1.5	1.6	0.6	1.0	0.6	2.0	0.7	4.6	0.7	0.2	0.3	1.1	0.0	1.6	2.3	1.5	2.0	0.8	0.7	1.5	0.7	2.3	2.8	PKS09	
2.2	5.8	3.7	4.5	2.5	7.1	6.6	6.6	5.5	n.p.	0.1	0.1	1.3	3.4	0.2	0.5	4.1	4.9	1.3	n.p.	3.0	5.7	3.4	2.5	0.7	3.7	5.8	4.6	4.4	n.p.	PKS10	Fusarin C
0.4	1.1	0.6	1.2	0.1	1.2	3.8	3.2	1.2	n.p.	4.4	0.3	1.5	0.7	9.0	0.5	0.6	2.8	0.9	n.p.	0.3	0.0	0.0	0.1	5.1	0.0	0.1	0.0	0.0	n.p.	PKS11	Fumonisins
0.0	0.0	0.0	0.1	0.0	0.0	0.0	0.0	0.0	n.p.	0.0	0.0	0.0	0.0	0.0	0.0	0.0	0.0	0.0	n.p.	0.0	0.0	0.0	0.1	0.0	0.0	0.0	0.0	0.0	n.p.	PKS12	
6.1	4.4	2.2	7.6	2.7	5.2	4.2	2.7	n.p.	1.1	7.9	3.9	2.1	4.6	2.0	2.5	6.3	2.1	n.p.	6.8	3.6	4.6	3.2	4.0	1.7	4.8	4.4	4.8	n.p.	3.4	PKS13	Gibepyrones
0.0	0.0	0.0	0.0	0.0	0.0	0.0	0.0	0.0	n.p.	0.0	0.0	0.0	0.0	0.0	0.0	0.0	0.0	0.0	n.p.	0.0	0.0	0.0	0.0	0.0	0.0	0.0	0.0	0.0	n.p.	PKS14	
0.3	0.2	0.3	0.5	7.2	8.4	0.3	0.4	n.p.	n.p.	0.1	0.0	0.0	0.2	1.9	0.0	0.0	0.0	n.p.	n.p.	0.0	0.0	0.0	0.2	0.4	0.0	0.0	0.0	n.p.	n.p.	PKS16	
0.0	0.0	0.0	0.0	0.0	1.2	0.0	0.1	n.p.	n.p.	0.0	0.0	0.0	0.0	0.0	0.1	0.0	0.2	n.p.	n.p.	0.2	0.4	0.1	0.1	0.0	0.1	0.1	0.1	n.p.	n.p.	PKS17	
0.0	0.0	0.0	0.0	0.0	0.0	0.0	0.0	n.p.	n.p.	0.0	0.0	0.0	0.0	0.0	0.0	0.0	0.0	n.p.	n.p.	0.0	0.2	0.0	0.0	0.0	0.0	0.0	0.0	n.p.	n.p.	PKS18	
1.7	2.1	2.0	n.p.	2.7	2.2	2.0	n.p.	n.p.	n.p.	2.9	3.2	2.9	n.p.	3.3	2.9	3.5	n.p.	n.p.	n.p.	1.5	1.8	1.7	n.p.	1.3	1.7	1.6	n.p.	n.p.	n.p.	PKS19	Fujikurins
0.1	0.4	0.4	0.4	0.7	1.3	0.4	0.1	0.3	0.2	1.1	0.6	0.6	1.0	1.3	0.7	0.7	0.3	0.6	0.1	0.1	0.3	0.3	1.0	1.1	0.3	0.3	0.2	0.6	0.1	PKS20	
n.p.	n.p.	n.p.	n.p.	n.p.	n.p.	n.p.	n.p.	n.p.	0.0	n.p.	n.p.	n.p.	n.p.	n.p.	n.p.	n.p.	n.p.	n.p.	0.0	n.p.	n.p.	n.p.	n.p.	n.p.	n.p.	n.p.	n.p.	n.p.	0.0	PKS39	Depudecin
n.p.	n.p.	n.p.	n.p.	0.1	n.p.	n.p.	n.p.	n.p.	n.p.	n.p.	n.p.	n.p.	n.p.	0.1	n.p.	n.p.	n.p.	n.p.	n.p.	n.p.	n.p.	n.p.	n.p.	0.0	n.p.	n.p.	n.p.	n.p.	n.p.	PKS40	
n.p.	n.p.	n.p.	n.p.	0.0	n.p.	n.p.	n.p.	n.p.	n.p.	n.p.	n.p.	n.p.	n.p.	0.0	n.p.	n.p.	n.p.	n.p.	n.p.	n.p.	n.p.	n.p.	n.p.	4.1	n.p.	n.p.	n.p.	n.p.	n.p.	PKS51	
1.3	5.2	6.3	3.5	3.5	0.0	7.9	0.6	0.8	1.9	2.5	1.9	2.0	1.1	0.6	0.4	5.2	0.1	1.1	1.5	0.3	0.3	1.1	2.7	0.2	0.2	5.6	0.2	0.2	3.9	STC1	
2.4	2.4	2.1	2.4	2.1	1.9	2.7	2.3	3.1	2.6	2.0	1.9	2.0	2.6	2.1	2.2	2.2	1.9	2.3	3.0	2.4	2.0	2.2	3.0	2.4	2.1	1.9	1.9	2.0	2.4	STC2	
0.0	0.0	0.0	0.1	0.0	0.0	0.0	0.0	0.0	0.0	0.5	0.4	0.0	0.1	0.1	0.8	0.6	0.0	0.8	0.0	0.1	0.0	0.1	0.0	0.6	0.4	0.0	0.0	0.0	0.1	STC3	Eremophilene
0.1	2.6	1.0	2.5	0.3	4.3	1.1	1.6	3.3	0.4	0.0	0.2	0.7	2.2	0.9	0.6	0.0	0.1	0.6	1.4	6.5	7.3	5.4	6.3	4.6	3.7	6.4	5.1	8.3	8.2	STC4	(+)-Koraiol
0.2	0.0	0.0	0.2	0.0	0.4	0.0	0.0	n.p.	0.0	0.0	0.0	0.0	0.4	0.0	0.0	0.0	0.0	n.p.	0.0	0.4	0.3	0.5	1.0	0.3	0.1	0.5	0.7	n.p.	2.3	STC5	Guaia-6,10(14)-diene
0.5	0.5	0.3	0.0	0.7	0.2	0.3	0.6	1.1	3.3	0.6	0.1	0.5	0.2	0.7	0.3	0.4	0.1	0.3	6.0	3.8	2.0	1.7	4.2	0.6	0.4	4.9	1.6	5.9	5.8	STC6	(-)-α-Acorenol
0.0	0.0	0.0	0.0	0.2	0.2	0.0	0.0	n.p.	0.1	0.0	1.9	2.2	1.1	1.5	0.2	1.5	2.0	n.p.	0.1	0.1	0.0	0.4	0.9	0.1	0.3	0.0	0.9	n.p.	0.2	STC7	
0.0	0.0	0.0	0.0	n.p.	0.0	0.0	0.9	0.0	0.0	0.0	0.0	0.0	0.0	n.p.	0.0	0.0	0.0	0.0	0.0	0.0	0.0	0.0	0.4	n.p.	0.0	0.0	0.0	0.0	0.8	STC8	
1.6	2.6	2.8	2.1	4.1	3.7	3.0	3.3	1.3	0.7	0.5	0.7	0.5	1.6	1.0	0.8	1.3	0.7	2.1	1.3	1.7	2.6	1.4	2.4	0.9	0.5	1.3	0.8	1.2	1.2	STC9	
2.7	6.3	6.1	5.4	3.5	4.3	7.5	5.6	n.p.	4.5	1.3	4.1	3.7	4.2	2.6	2.5	4.2	3.4	n.p.	1.0	2.6	4.0	4.5	4.4	6.0	6.6	1.4	4.8	n.p.	2.9	TeTC1	Carotenoids
3.1	4.4	4.4	4.1	4.1	4.0	3.7	3.8	3.8	4.5	3.5	4.0	3.6	3.8	4.1	4.4	4.1	4.3	3.6	3.1	5.0	5.9	5.7	6.1	4.9	5.9	6.4	5.6	4.4	4.2	TrTC1	

The most prominent and species-specific SMs are the GAs causing the *F*. *fujikuroi*-specific *bakanae* disease of rice. Although some recently sequenced species of the FFC such as *F*. *mangiferae* and *F*. *proliferatum* contain one or even two GA gene clusters, these species produce either no or only very small amounts of GAs [43]. In this work, we examined whether all analyzed *F*. *fujikuroi* strains produce GAs, and whether the GA levels can be correlated to the virulence on rice.

**Fig 5:**
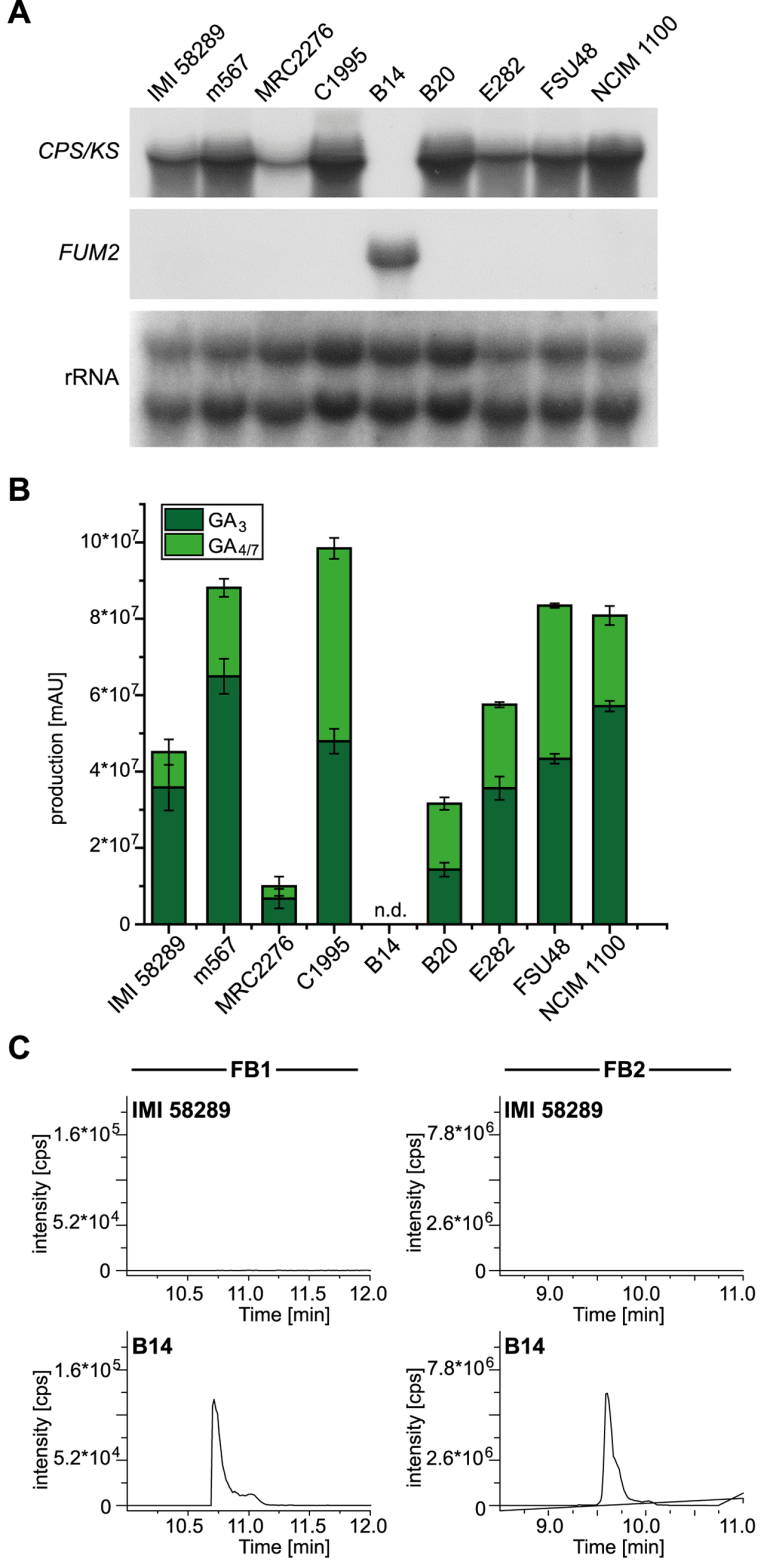
Gibberellic acid (GA) and fumonisin production and gene expression in the ten analyzed strains. A) GA (*CPS/KS*) and fumonisin (*FUM2*) gene expression studies by Northern blot analysis after 3 days of growth in synthetic medium with 6 mM glutamine. GA (B) and fumonisin (C) production levels after 7 days of growth in synthetic medium with 6 mM glutamine.

Previously, the regulation of GA biosynthesis has been extensively studied for strain IMI 58289. It has been shown that GA gene expression is strictly regulated by nitrogen availability in an AreA- and AreB-dependent manner [47–49]. To examine whether low nitrogen conditions are optimal for GA production also in the other isolates, we performed HPLC-DAD analysis of all strains and analysed the expression by Northern blot analysis in addition to RNA-seq data. Accordingly, we observed the highest GA yields and GA gene expression levels under low nitrogen conditions for eight of the nine *F*. *fujikuroi* strains. The only exception among the *F*. *fujikuroi* isolates is strain B14 which showed no production of GAs and no visible expression of the GA genes despite the presence of the complete GA gene cluster in the genome (Table 5A and 5B; Fig 5A and 5B). Besides the very low expression of GA genes, strain B14 differs from the other strains by high expression of fumonisin biosynthetic genes under low nitrogen conditions (Table 5A, Fig 5A and 5C). Recently, we have shown that the fumonisin gene cluster in *F*. *fujikuroi* IMI 58289 is almost silent. Consequently, only very low amounts of fumonisins are produced in comparison to *F*. *verticillioides*. In *F*. *fujikuroi* IMI58289, the genes are only expressed and fumonisins are only produced, when the cluster-specific TF gene *FUM21* is constitutively and strongly expressed [50]. Here, similar results were observed for eight of the nine *F*. *fujikuroi* isolates (except for B14) which all produce either no or only very small amounts of fumonisins and showed very low expression levels.

In addition, B14 is the only isolate producing fujikurins under alkaline and low nitrogen conditions (Table 4). Previously, the fujikurin gene cluster (PKS19) was shown to be silent under all conditions tested in strain IMI 58289, and the products have been identified only after simultaneous over-expression of both the cluster-specific TF gene and the PKS19 gene itself [15,34].

In most of the strains, fusarins, fusaric acid, and apicidin F (except for B14) were only produced under high nitrogen conditions (60 mM glutamine) in accordance with the higher expression levels of the corresponding biosynthetic genes. The genes of the beauvericin cluster were recently shown to be silent under all conditions tested and activated only after deletion of the histone deacetylase gene *HDA1* and knock-down of the histone methyltransferase gene *KMT6* in strain IMI 58289 [33,38]. Most of the other strains analyzed here showed no or very low expression levels and no beauvericin production. The only exception was strain B20 that highly expressed the biosynthetic genes under both nitrogen conditions (low and high amounts of glutamine) and produced high levels of beauvericin at high nitrogen (Table 4; Table 5A and 5B) suggesting that the chromatin status around the beauvericin cluster differs in B20.

### Virulence assay on rice

To examine whether the isolates differ in their virulence on rice, and whether the different levels of GAs or the strain-specific production of other SMs (e.g. fumonisins in B14) correleate with the extent of symptom development, we performed assays with both germinating seeds and rice seedlings.

To determine the ability of all ten strains to impair the seed germination, rice seeds were soaked for 18 h in spore suspensions of the respective strains. The percentage of germinated seeds was counted after 14 days post inoculation (dpi). Strains B14 and B20 induced a high percentage of seedling death indicating a high potential to kill the host seedlings (S4 Table). As B14 does not produce much GAs, the aggressiveness of this strain cannot be caused by these phytohormones.

**Fig 6:**
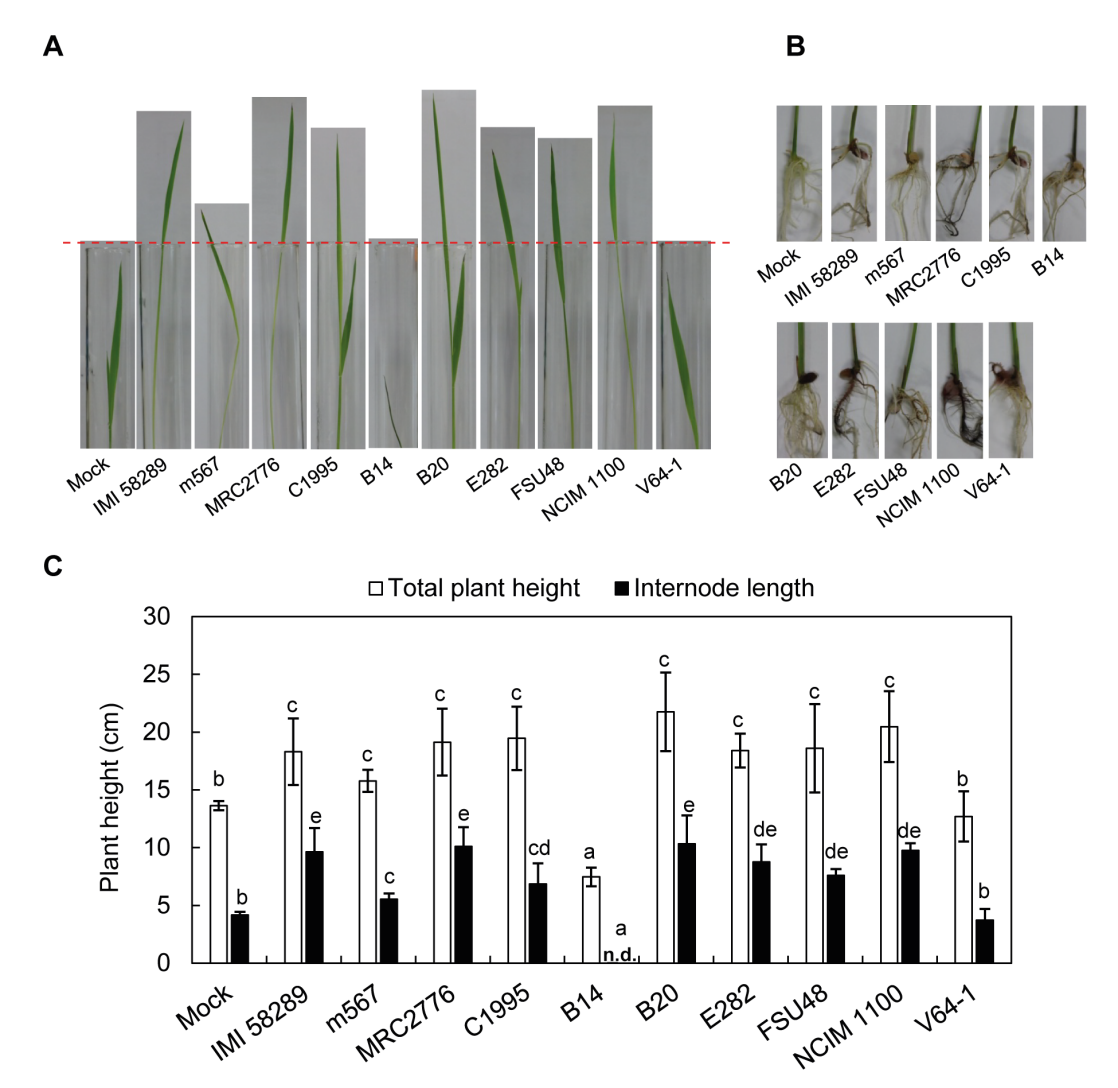
Pathogenicity assay of the *F*. *fujikuroi* and *F*. *oxysporum* isolates. (A) Symptoms in the rice stems that were inoculated with the fungal isolates and water only (control). The uppermost stem level of the rice seedling with control treatment is indicated by a thin dotted bar. (B) Symptoms in the rice roots after pathogen infection. (C) The plant heights and internode lengths of pathogen-infected rice seedling at 6 dpi. Error bars show standard deviations. The same letter above bars indicates no significant difference. n.d., not detected.

Next, we performed a rice seedling assay to compare the virulence of the different isolates to assess their ability to induce *bakanae* symptoms. The surface-sterilized seeds were first germinated, and the young healthy seedlings were then inoculated with spores. All strains except for B14 and *F*. *oxysporum* V64-1 (outgroup) induced the formation of slender, elongated and yellowish stems (Fig 6). Wheras V64-1-infected seedlings behaved like the water control, B14-infected seedlings were stunted and showed withering instead of the typical *bakanae* symptoms. The total seedling length and the length of the internodes were smaller than, or comparable to uninfected seedlings (water control). Previously, it has been reported that heavily infected seedlings can also be stunted and can show severe crown and root rot [51]. The type of symptoms and severity of disease depends on the fungal isolate and is thought to be affected by the proportions of GA and fusaric acid produced by the fungus, which potentially cause elongation of the plants or stunting, respectively [52,53]. However, this assumption has never been proved experimentally.

### *In planta* expression of SM gene clusters and product analysis

To examine which of the SM gene clusters are similarly expressed in rice in all isolates, and which of them are specifically expressed or not expressed in strain B14, and therefore could be relevant for disease symptoms (especially stunting), we performed RNA-seq for all isolates also from rice seedlings at 7 dpi (Table 5C). The expression patterns were compared with those under *in vitro* conditions at high (60 mM glutamine) and low (6 mM glutamine) nitrogen (Table 5A and 5B).

In general, the GA biosynthetic genes (shown for the key enzyme gene *CPS*/*KS*) are the most highly expressed genes in rice roots except for B14 in which they are hardly expressed (Table 5C).

To check whether the expression profiles of SM genes correspond to the *in planta* production levels, we also performed SM analysis of infected rice plants by HPLS-FTMS at 7 dpi. In accordance with gene expression, the *in planta* GA production levels also differed between the isolates: B14-infected rice plants contained no detectable GA amounts in rice compared to the *bakanae* strains (Table 6). The most obvious difference of strain B14 compared to the other isolates was the high expression of the fumonisin genes resulting in significant levels of fumonisins (FB_1_ and FB_2_) *in planta*, similar to those observed *in vitro* (Table 5C; Table 6). Furthermore, while all isolates showed similar expression patterns for fusaric acid biosynthetic genes (*PKS6*; *NRPS34*) and similar fusaric acid production levels (Table 5C, Table 6), B14 was the only strain with low expression of the fusarin C genes. As expected, no fusarins were detectable in rice roots inoculated with this strain (Table 6).

**Table 6:** Analyses of secondary metabolites in rice after seven dpi.

Strain	BIK	FSR	FSA^a^	APF	BEA^a^	FB_2_	FB_1_	FusC	TST	FUJ	GA_3_
**IMI 58289**	**+**	**+**	**+**^**b**^	**+**	**+**^**b**^	**-**	**-**	**+**	**-**	**-**	**++**
**m567**	**+**	**++**	**++**	**++**	**+**	**-**	**-**	**++**	**-**	**-**	**+++**
**MRC2276**	**+++**	**++**	**++**	**++**	**++**	**-**	**-**	**++**	**-**	**-**	**++**
**C1995**	**+**	**+**	**++**	**++**	**++**	**-**	**-**	**++**	**+**	**-**	**++**
**B14**	**+**	**+**	++	n.p.	**++**	**+++**	**+++**	**-**	**++**	**-**	**-**
**B20**	**++**	**++**	**++**	**++**	**+++**	**-**	**+**	**+++**	**+++**	**-**	**++**
**E282**	**++**	**+++**	**++**	**+**	**+**	**-**	**+**	**++**	**-**	**-**	**++**
**FSU48**	**+**	**+**	**+++**	**+**	**++**	**-**	**-**	**++**	**-**	**-**	**++**
**NCIM 1100**	**++**	**+**	**++**	**+++**	**+**	**-**	**-**	**++**	**-**	**-**	**++**
**V64-1**	**++**	**++**	**+**	**+**	**++**	**-**	**+**	**-**	**-**	**-**	**-**
**water control**	**-**	**-**	**-**	**-**	**-**	**-**	**-**	**-**	**-**	**-**	**-**

**+++**: 90–100% of the max. value; **++**: 10–90% of the max. value; **+**: <10%; -: not detectable; n.p. cluster not present; a: diluted 1/10, except for b; BIK–bikaverin, FSR–*O*-methylfusarubin, FSA–fusaric acid, GPY–gibepyrone A, APF–apicidin F, BEA–beauvericin, FB_2_ –fumonisin B_2_, FB_1_ –fumonisin B_1_, FusC–fusarin C, TST–trichosetin, FUJ–fujikurin A, GA_3_- –gibberellic acid GA_3_

In addition, the yet uncharacterized PKS51 gene cluster, which is only present in B14, was exclusively expressed *in planta*, suggesting that the unknown product of this gene cluster might play a role during infection (Table 5C). Besides B20, B14 also gave high expression for beauvericin genes, but only low expression of gibepyrone (PKS13) and acorenol (STC6) biosynthetic genes.

In conclusion, the very low expression of GA genes and the lack of detectable GA levels in B14-infected rice seedlings after 7 dpi are most likely responsible for the absence of *bakanae* symptoms in this isolate. Instead, the high levels of fumonisins which are exclusively produced only in this isolate, may overrule the growth-stimulating effect of the GAs and cause stunting/withering.

### Do the fumonisins, fusaric acid and/or the PKS51 product cause the stunting symptom development of *F*. *fujikuroi* B14?

To find out whether one of the gene clusters that are specifically expressed in B14 during infection on rice (Table 5C) might indeed cause the stunting effect of this isolate, the key enzyme-encoding genes for fumonisins (*FUM1* = *PKS11*), and the yet uncharacterized B14-specific *PKS51* gene were deleted in this strain. In addition, we also deleted the key gene for fusaric acid biosynthesis (*FUB1* = *PKS6*) due to the speculation that fusaric acid production might cause the stunting pathotype [52].

**Fig 7:**
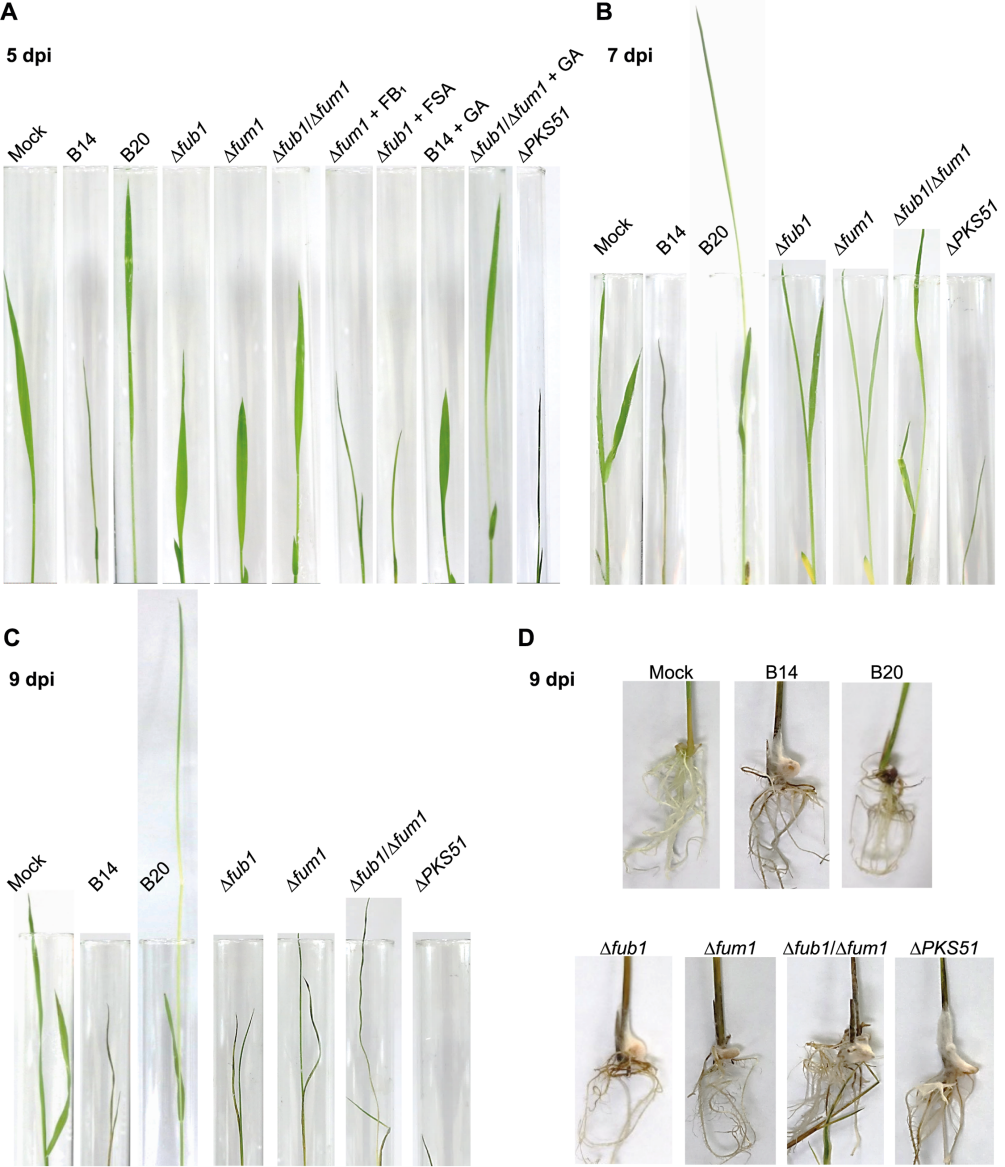
Shoot and root growth of rice seedlings 5, 7, and 9 days post inoculation (dpi) of *F*. *fujikuroi* B14 and gene deletion strains dervied from B14. (A-C) Shoot growth. (D) Root growth. (A) Shoot and root growth 5 dpi with or without exogenous supply of fumonisin FB_1_ (1 μM), fusaric acid (FSA; 10 μM) or GA_3_ (10 μM). (B) Shoot growth 7 dpi. (C) Shoot growth 9 dpi. Mock: neither fungal inoculation nor toxin treatement; B14 (stunting pathotype) and B20 (*bakanae* pathotype) wild-type strains; Δ*fub1* (*PKS6*, fusaric acid) deletion strain; Δ*fum1* (*PKS11*, fumonisins) deletion strain; Δ*fub1/*Δ*fum1* double deletion strain; Δ*PKS51* (*PKS51*, unknown product).

The 5-day-old healthy rice seedlings were soaked in the conidial suspension of B14 or the mutant strains. At 5 dpi, the B14-infected seedlings were already stunted compared to the water control and showed withering symptoms which were even more obvious at 7 dpi and 9 dpi (Fig 7A). In contrast, the Δ*fum1* and Δ*fub1* strains of B14 appeared healthy at 7 dpi and caused delayed withering symptoms at 9 dpi only. The roots of all infected seedlings were heavily colonized with fungal mycelia at 9 dpi (Fig 7D). The double deletion mutants of B14 lacking both *FUM1* and *FUB1* behaved like the mock control and did not induce stunting at 5 dpi (Fig 7A) or occasionally upto 9 dpi. However, in some cases, they caused a similar delayed disease development as the single deletion strains at 9 dpi (Fig 7B and 7C). The add-back strains, which were generated by introducing the native copy of *FUM1* and *FUB1* into Δ*fum1* and Δ*fub1* mutants, respectively, caused typical stunting/withering symptoms similar to B14 (S4A Fig). The delay of stunting/withering symptom development by Δ*fum1* and Δ*fub1* strains indicates that the production of fumonisins and/or fusaric acid, in combination with the non-detectable levels of GAs, play an important role for the development of this specific pathotype in B14.

For comparison, the genes *FUM1* and *FUB1* were also deleted in strain B20 (*bakanae* strain). Unlike the B14-derived mutants, all of the gene deletion strains of B20 we examined caused typical *bakanae* symptoms (elongated, slender and chlorotic shoots), as did B20. No withering of the seedlings or mycelial colonization on roots were visible suggesting that the absence of fumonisin production by B20 and B20-1 derived mutants protect the rice seedlings from development of B14-like symptoms (S5 Fig).

Surprisingly, rice seedlings inoculated with the Δ*PKS51* mutant strains showed an earlier and more severe symptom development compared to those with B14. The stunting/withering symptoms were clearly shown already at 5 dpi (Fig 7A). The more severe symptoms caused by the Δ*PKS51* strains may suggest a possible role of the PKS51 product as an avirulence determinant. A similar role was described for the product of the *Magnaporthe grisea* PKS-NRPS gene *ACE1* (Avirulence Conferring Enzyme1) which is also specifically expressed only on rice. Its yet unknown product is probably recognized by rice cultivars carrying a specific resistance gene [54,55]. However, further investigations will be needed to show whether the product of PKS51 acts in a similar way in *F*. *fujikuroi* strain B14.

To further study the impact of these SMs on disease symptom development, fumonisin FB_1_, fusaric acid, or GA_3_ were exogenously supplied to rice seedlings infected with wild-type or mutant strains (Fig 7A). Addition of FB_1_ and fusaric acid to rice seedlings infected with Δ*fum1* and Δ*fub1*, respectively, restored the WT phenotype and resulted in stronger withering symptoms compared to the deletion strains without the toxins. However, the even more reduced virulence of the double deletion strain (Δ*fum1/*Δ*fub1*) was not clearly complemented by exogenous supplies of both fumonisin and fusaric acid (Fig 7A). Interestingly, exogenous supply of GA_3_ did not cause *bakanae* symptoms on rice seedlings inoculated with the wild-type B14 strain, while addition of GA_3_ to the Δ*fum1/*Δ*fub1* double deletion strain caused stem elongation of rice seedlings at 5 dpi, similar to the *bakanae* symptom caused by B20 (Fig 7A). Therefore, conversion of B14 into a *bakanae* pathotype by addition of GAs was only possible after deleting the key genes for the production of fumonisins and fusaric acid.

Furthermore, exogenous supply of culture filtrate from strain B14 to seedlings infected with B14, B20 or B20 Δ*cps*/*ks* caused stunting and withering symptoms while the culture filtrate of the B14 Δ*fum1*/Δ*fub1* mutant caused milder symptoms (S4B and S4C Fig). Addition of culture fluid to B14-infected seedlings resulted in even more severe stunting than B14 alone (S4B Fig). Exogenous supply of B14 culture filtrate to seedlings inoculated with B20 or the GA-deficient B20 Δ*cps/ks* mutant revealed severe stunting symptoms in both cases irrespective of the ability to produce GAs (S4C and S4D Fig). These data support our suggestion that fumonisins and fusaric acid play an important role for symptom development.

To further investigate the role of fumonisins for causing stunting and withering, we performed the rice seedling pathotest with two *F*. *verticillioides* strains from corn which were shown to produce high amounts (more than 3,000 μg/g) of fumonisins and no GAs in rice seedlings. Both *F*. *verticilloides* isolates (Os35 and Os40) [56] caused withering at 7 dpi although the plants were not stunted compared to those of the mock control (S6A Fig). In addition, mycelia of both *F*. *verticillioides* strains colonized the roots of infected rice seedling as much as those of B14 (S6B Fig). This strong root colonization was not observed for roots infected with B20. Previously, it has been already reported that B14 triggered severe inhibition of root growth, and that its own growth rate in rice roots was more than 4 times higher compared to that of B20.

Taking together the results of *FUM1* and *FUB1* deletion, exogenous addition of pure toxins or culture filtrate of B14 to rice seedlings inoculated with wild-type or mutant strains, and the pathotests with GA-deficient, highly fumonisin-producing *F*. *verticillioides* isolates provide strong indications that the biosynthesis of both fumonisins and fusaric acid and the lack of GA biosynthesis in B14 play cruicial roles for causing the stunting/withering phenotype on rice seedlings. However, additional factors are probably involved in inducing the stunting symptoms.

### Comparisons of TF-encoding genes between B14 (stunting) and B20 (*bakanae*) strains of *F*. *fujikuroi*

Besides SMs, stunting/withering might be caused also by the different sets of TFs present in the genome of B14 and the *bakanae* strains, or by different expression levels of TF-encoding genes in rice. Therefore, we compared the expression levels of TF-encoding genes between B14 and the other eight *F*. *fujikuroi* isolates (S5A Table). There are 37 genes which are present in most of the nine genomes and which were specifically up-regulated during infection of rice (S5B Table). B14 had slightly higher expression levels only for three of them: *FFB14_03090*, *FFB14_05980* and *FFB14_01631*. In addition, B14 has 28 strain-specific TFs which are not present in the genomes of the other strains (S5C Table). The most highly expressed gene *in planta* was *FFB14_06367* encoding the pathway-specific TF of the putative PKS51 gene cluster. The high expression of *PKS51* and the adjacent genes, including the TF-encoding gene, supports our assumption that this unique gene cluster is involved in determining the severity of disease symptom development.

### Population analyses revealed the segregation of field isolates into the two different pathotypes

**Fig 8:**
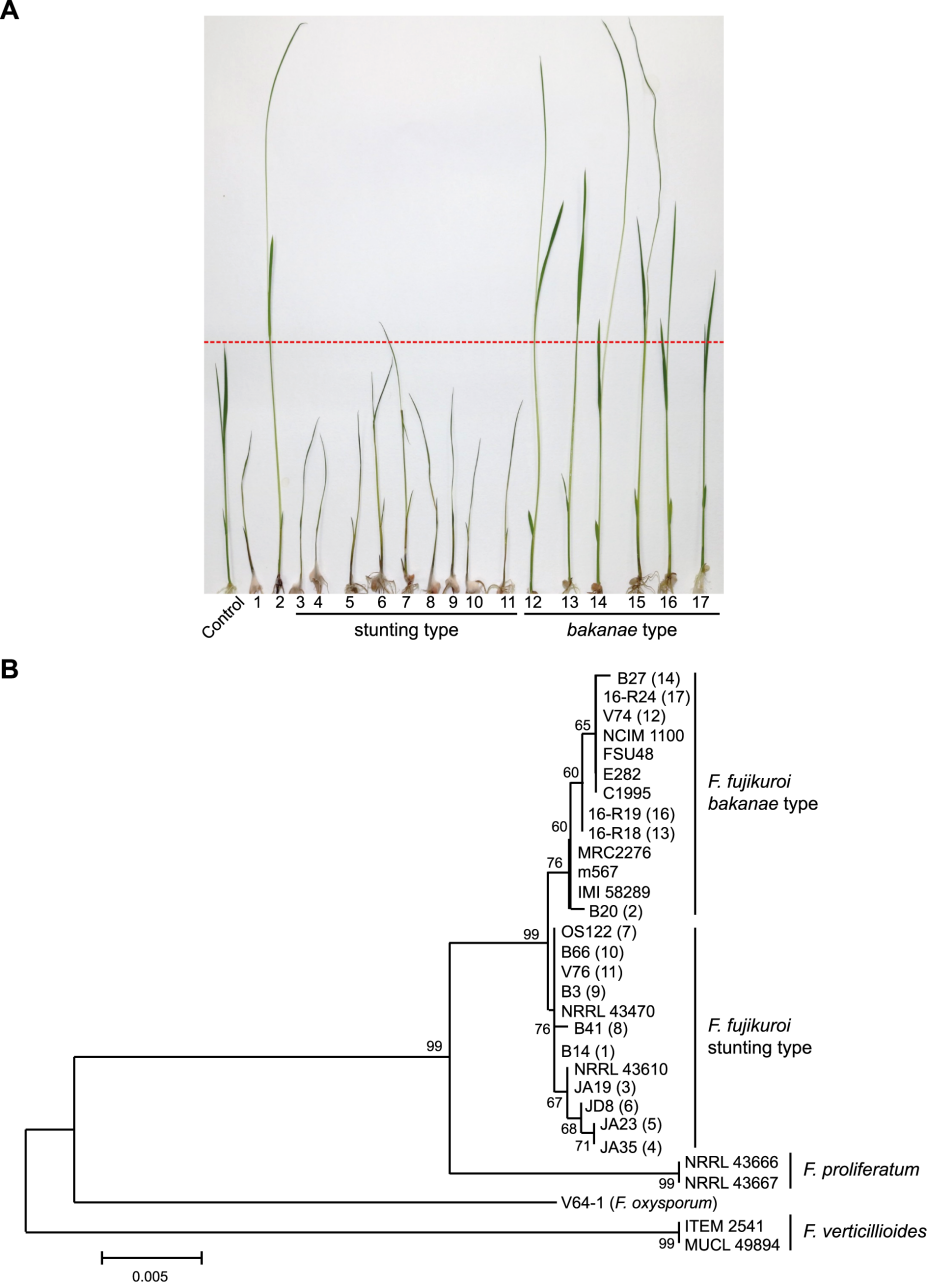
Population analysis of field isolates in Korea. (A) Rice seedlings 9 dpi of *F*. *fujikuroi* field isolates obtained from Korea. Control: no fungal inoculation, 1: B14, 2: B20, 3: JA19, 4: JA35, 5: JA23, 6: JD8, 7: OS122, 8: B41, 9: B3, 10: B66, 11: V76, 12: V74, 13: 16-R18, 14: B27, 15: B17, 16: R19, 17: 16-R24. Pathogenicity test with 15 additional isolates revealed a clear separation between the *bakanae* and stunting pathotype. (B) A phylogenetic tree constructed by the NJ method using the nucleotide sequences of combined *TEF1* and *RPB2* from the two pathotypes of field isolates, determined by pathogenicity test (A), and from the worldwide isolates used in this study. The phylogenetic tree supports the separation of the two pathotypes into two different phylogenetic subclades.

Because B14 was the only isolate causing the stunting pathotype among the analyzed ten strains, we attempted to determine how often this pathotype can be found in rice fields. Therefore, we inoculated rice seedlings with 15 field isolates, which were collected from rice grains and air above rice paddy fields in Korea between 2014 and 2016. Among the 15 isolates, we identified additional nine field isolates causing stunting and early withering symptoms similar to B14 while six isolates caused typical *bakanae* symptoms (Fig 8A).

To determine whether these isolates can be phylogenetically distinguished from each other, we generated a phylogenetic tree using the nucleotide sequences of the *RPB2* and *EF1A* genes [57] from the new field isolates, the ten isolates used in this study, and some closely related *Fusarium* species of the FFC. The *F*. *fujikuroi* clade, which was clearly separated from those of other closely related species such as *F*. *proliferatum* and *F*. *verticillioides*, contained two strongly supported subclades (with 76% bootstrap support: BS). Interestingly, the subclade with B14 consists of all of the field isolates causing stunting symptoms, while the other subclade contained B20 and the other *F*. *fujikuroi* strains used in this study and all *bakanae*-type field isolates (Fig 8B). This result indicates that the two pathotypes of *F*. *fujikuroi* exist as phylogenetically distinct groups within the population.

To examine if there is a correlation between the pathotype and the presence of one of the SM clusters specific to B14 or B20 (*PKS51* for B14, unknown product, and *NRPS31* for B20, apicidin F) among the field isolates of each pathotype, we performed a PCR amplification using primer sets derived from *PKS51* (unknown product) and *NRPS31* (apicidin F), respectively. The *PKS51-*specific primer set amplified a fragment from all of the stunting-type isolates examined including B14, but not from the *bakanae*-type isolates. Similarly, the *NRPS31*-specific primer set amplified a fragment only from *bakanae*-type isolates examined (S7A Fig).

Based on the correlation between the phylogenetically distinct pathotypes on the one hand, and the presence of either the *PKS51* or the apicidin F (*NRPS31*) gene clusters, we examined additional field isolates from Korea using these primer sets. Among a total of 151 isolates examined, the B14-specific PCR amplification pattern was found in 69% of rice isolates, 100% of corn isolates, and 85% of airspora isolates. A phylogenetic tree generated from all of these isolates revealed a clustering of the stunting-type and *bakanae*-type isolates similar to that for the field isolates examined by pathogenicity tests (S7B Fig). However, it is currently unclear why the stunting-type population is predominant among the field isolates in Korea.

To gain more information on the SM profile of these new stunting-type isolates, we examined the expression levels of the key genes of GA (*CPS*/*KS*) and fumonisin (*FUM1*) biosynthesis in the field isolates grown in liquid culture with 6 mM glutamine by use of quantitative real-time PCR (qPCR). The *CPS*/*KS* transcript levels from all the stunting-type isolates examined were similar to or even lower than that in B14, while those from all the *bakanae*-type isolates, including B20, were 8- to 24-fold higher than those in the stunting-type strains (S8A Fig). Furthermore, all stunting-type isolates showed clear expression of *FUM1* in contrast to the *bakanae* isolates (shown for B20) (S8B Fig). These data indicate that both subgroups of isolates differ in a whole set of characteristic features (presence of either the *PKS51* or *NRPS31* genes, expression of either GA or fumonisin genes) which correlate with either the stunting or the *bakanae* pathotype. We also examined the production levels for both SMs in some of the new B14-like strains *in vitro* and *in planta*. As expected, the stunting-type isolates produced no or 10 to 15 times less GAs than the *bakanae* strain IMI 58289, while only the stunting-type strains produced fumonisins in submers cultures (S9A and S9B Fig). The analysis of GAs and fumonisins in rice roots and shoots revealed no GAs at all in seedlings infected with stunting-type isolates, while only the latter produced significant amounts of fumonisins *in planta* (S9C and S9D Fig). A similar correlation between low GA and high fumonisin levels on the one hand, and a pathotype called “dwarfism” on the other hand, was also described for the Italian *F*. *fujikuroi* isolate CSV1 [58].

In conclusion, we provide genome sequences of eight new *F*. *fujikuroi* isolates and one *F*. *oxysporum* isolate, all collected in different rice growing regions worldwide. We show that all these strains differ in genome and chromosome size, number of genes in major gene families such as TFs, transporters, SM biosynthetic genes and others. In addition, the isolates differ in colony morphology, pigmentation and the number and type of asexual spores (micro- and/or macroconidia). Major differences were identified in subtelomeric regions of the chromosomes where several SM gene clusters are located. Besides the differences in the presence of gene clusters, we observed variations in the ability to express SM genes and to produce the respective metabolites under *in vitro* and *in planta* (rice) conditions. Among the nine *F*. *fujikuroi* isolates analyzed, eight cause typical *bakanae* symptoms on rice seedlings due to their ability to produce GAs. Only one isolate, B14, does not cause elongation of infected plants, but instead causes stunting combined with early withering of rice seedlings. This isolate is the only one which does not produce GAs under *in vitro* and *in planta* conditions. Instead, B14 produces high amounts of fumonisins both under *in vitro* and *in planta* conditions. Furthermore, it is the only strain containing a putative gene cluster with *PKS51* as key gene which is highly expressed in rice. To examine the determinant(s) of the stunting/withering pathotype, several key enzyme-encoding candidate genes were deleted in B14. The data demonstrate that the formation of fumonisins, and probably also fusaric acid, on the one hand, and the lack of GA production on the other hand, contribute to the stunting/withering pathotype, because the deletion of the respective key genes resulted in reduced virulence. Furthermore, the unknown PKS51 gene cluster seems to produce a SM which acts as an attenuator of disease, because the deletion of the *PKS51* gene caused early withering of infected seedlings. Examination of more field isolates from Korean rice fields revealed a correlation between the pathotype and the ability to produce either fumonisins or GAs which is supported by the clear separation into two distinct phylogenetic clades.

## Material and methods

### Fungal and bacterial strains

The fungal strains used in this work and their origin are shown in Table 1. Strain IMI 58289 was derived from Commonwealth Mycological Institute, Kew, United Kingdom. Strains m567 and FSU48 were provided by the Fungal Stock Center at the University Jena, Germany, C1995 by J.F. Leslie, Kansas State University, E282 by S. Tonti, University Bologna, Italy, MRC2276 by W.C.A. Gelderblom, South Africa, and NCIM 1100 was provided by the National Collection of Industrial Microorganisms, India. Strains B14 and B20 were provided by S.-H. Yun, Korea. Strain V64-1 was kindly provided by T. Kyndt from the University Ghent, Belgium.

*Escherichia coli* strain Top10 F’ (Invitrogen, Groningen, The Netherlands) was used for plasmid propagation. The uracil-auxotrophic *Saccharomyces cerevisiae* FGSC 9721 (FY 834) was provided by the Fungal Genetics Stock Center (Kansas State University) and used for yeast recombination cloning.

### Genome sequencing and assembly

*F*. *fujikuroi* B20: Illumina TrueSeq genome sequencing by TheragenEtex, Suwon, Korea. The assembly was performed using Celera Assembler version 7.0 [59], ‘overlap minimum length’ set to 150 bases. The assembly resulted in 318 scaffolds with a 21-fold coverage of the TrueSeq large reads.

For all other strains sequencing was carried out by shot gun sequencing of an 8 kb library with paired end 100 bp read length using Illumina HiSeq 2000 by Eurofins MWG Operon, Germany. The assemblies were performed by ALLPATHS-LG [60] and the scaffolds were error corrected by mapping all Illumina shotgun paired-end data and further scaffolded using SSPACE [61] (Table 2). The data on the new genomes, including annotation, was submitted to the European Nucleotide Archive, study accession PRJEB14872 available at: http://www.ebi.ac.uk/ena/data/view/PRJEB14872. Sample accession numbers are listed in Table 1. RNA-seq data are available at: https://www.ncbi.nlm.nih.gov/gds/?term=GSE89480.

### Structural annotation

Draft gene models for all genomes were generated by three *de novo* prediction programs: 1) Fgenesh [62] with different matrices (trained on *Aspergillus nidulans*, *Neurospora crassa* and a mixed matrix based on different species); 2) GeneMark-ES [63] and 3) Augustus [64] with *Fusarium* ESTs and RNA-seq based transcripts as training sets. Annotation was aided by exonerate [65] hits of protein sequences from *F*. *fujikuroi* IMI 58289 and *F*. *oxysporum* 4287 to uncover gene annotation gaps and to validate *de novo* predictions. Transcripts were assembled on the RNA-seq data sets using Trinity [66]. The different gene structures and evidences (exonerate mapping, RNA-seq reads and transcripts) were visualized in GBrowse [67] allowing manual validation of coding sequences with a focus on SM cluster genes and other genes of interest. The best fitting model per locus was selected manually and gene structures were adjusted by splitting or fusion of gene models and redefining exon-intron boundaries if necessary. tRNAs were predicted using tRNAscan-SE [68]. The predicted protein sets were searched for highly conserved single (low) copy genes to assess the completeness of the genomic sequences and gene predictions. Orthologous genes to all 246 single copy genes were searched for all proteomes by BLASTp comparisons (eVal: 10^−3^) against the single-copy families from all 21 species available from the FunyBASE [21]. Additionally, the proteomes were searched for the 248 core-genes commonly present in higher eukaryotes (CEGs) by BLASTp comparisons (eVal: 10^−3^) [20]. We also used BUSCO Version 3.0.1 in the Ubuntu virtual machine with the lineage specific profile library Sordariomyceta_odb9 (3.725 BUSCO groups), downloaded from http://busco.ezlab.org. The analysis was performed in gene set (protein) assessment mode running the python script run_BUSCO.py [22]. All genomes were analyzed using the PEDANT system [69]. To avoid misleading ortholog information based on similarity and bi-directional best hits, QuartetS [70] was applied to retrieve a reliable ortholog matrix which was used for all comparative representations.

### Phylogenetic tree analyses

The phylogenetic tree of *Fusarium* species was calculated based on the protein sequences of 5,181 single copy genes that are shared among all analyzed species. Orthologs of the sequences were aligned separately using MAFFT [71]. After that, we concatenated the alignments and removed columns with gaps using Gblocks [72]. The evolutionary history was inferred using the Maximum Likelihood method PhyML [73] with default parameters and the amino acid substitution model LG. Branch support was tested using approximate likelihood ratio test (aLRT) based on the Shimodaira-Hasegawa-like (SH-like) procedure [74]. The tree is drawn to scale, with branch lengths measured in the number of substitutions per site.

We calculated single copy genes in clustering proteins of all genomes and selecting clusters with exactly one representative from each genome. Protein clusters were calculated using usearch [75] (e-value cutoff: 0.01) and mcl [76] (inflation value: 2).

For phylogenetic analysis of field isolates of *F*. *fujikuroi* in Korea, *TEF1α* and and *RPB2* were amplified from fungal genomic DNAs using the primer sets fRPB2-7cF/fRPB2-11aR [77] and EF1/EF2 [78], respectively (S6 Table). All nucleotide sequences from PCR products were edited with Lasergene (ver. 6.0; DNASTAR, Madison, WI, USA) and aligned using ClustalW [79]. Maximum parsimony (MP), neighbor-joining (NJ), and unweighted pair group method with arithmetic mean (UPGMA) analyses were performed using MEGA (ver. 4.02) with 1,000 bootstrap replications.

### Contour-clamped homogeneous electric field (CHEF)-gel analysis

Protoplasts from *Fusarium* strains were prepared as described previously. The protoplast suspension was mixed with 1.2% InCert agarose (Lonza Group AG, Basel, Switzerland) and then loaded on a CHEF gel as described in [80]. Chromosomes of *Schizosaccharomyces pombe* and *S*. *cerevisiae* were used as a molecular size marker (Bio-Rad, Munich, Germany).

### *In silico* identification of secondary metabolite clusters

To identify SM clusters in each genome, the InterPro scan results of the PEDANT analysis were used as described [81]. Essentially, predicted proteins with homology to a domain of a signature SM core enzyme (e.g. PKS, NRPS, TC or DMATS) were considered a marker for a gene cluster. A cluster was verified if any neighboring genes with homology to typical SM cluster enzymes, like P450 monooxygenases, oxidases, methyltransferases, MFS or ABC transporters or TFs, were identified. The extent of each putative gene cluster was then adjusted by comparison to previously published data and to homologous clusters in other *Fusarium* species.

### Cultivation methods

For SM production experiments and RNA preparation under *in vitro* conditions, strains were first cultivated for 3 days in 300 mL Erlenmeyer flasks with 100 mL Darken medium [82] on a rotary shaker at 180 rpm at 28°C. 500 μL of this culture were then used to inoculate 100 mL of ICI (Imperial Chemical Industries, UK) media [83] containing either 6 mM glutamine, 60 mM glutamine, or 6 mM NaNO_3_ for 3 days (RNA extraction) or 7 days (SM analysis), respectively. For RNA extraction, mycelia were flash-frozen with liquid nitrogen prior to lyophilization. For *in planta* expression studies by RNA-seq, lyophilized roots of infected rice plants were used for RNA preparation.

### Plant material for RNA sequencing

For the generation of infected rice samples for transcriptome analysis by RNA-seq, rice seeds of the cultivar GSOR 100, Nipponbare, and Dongjin were used. The seeds of the former two were provided by Genetic Stocks-Oryza (GSOR) Collection, USDA ARS Dale Bumpers National Rice Research Center, Hwy, Arkansas, USA.

### RNA isolation, RNA sequencing and quantification

Total RNA was extracted from mycelia grown for 3 days in liquid ICI media (containing either 6 or 60 mM glutamine) and from infected rice seedlings after 7 dpi using TRIzol Reagent (Life Technology, Karlsruhe, Germany) and purified using an RNeasy Plant MinElute Cleanup Kit (Qiagen, Hilden, Germany). The quality of DNase-treated RNA (28S:18S > 1.0; RIN≥ 6.5; OD_260/280_ ≥1.8; OD_260/230_ ≥ 1.8) was determined using an Agilent Bioanalyzer. The high quality RNA was sent to BGI Tech Solutions Co., Limited (Hong Kong) for library construction and sequencing by Illumina HiSeq2000 technology.

RNA-seq reads were mapped on the reference genome using tophat2 (v2.0.8). The interval for allowed intron lengths was set to min 20 nt and max 1 kb [84–86]. We used cufflinks to determine the abundance of transcripts in FPKM (fragments per kilobase of exon per million fragments mapped) and calculated differentially expressed genes using cuffdiff [85,86]. The gene models were included as raw junctions. Genes with a minimum of fourfold increase or decrease in expression (|log2 of the FPKM values +1| ≥ 2) between the two experimental conditions were considered as regulated. The RNA-seq data has been deposited in NCBI's Gene Expression Omnibus [87] and are accessible through GEO Series accession number GSE89480.

### Generation of vectors for targeted gene deletions and complementations

The DNA constructs for deletion of *FUM1* (FFB14_08440, FFB20_01984), *FUB1* (FFB14_01651FFB20_13404), and *PKS51* (FFB14_06372) from the genomes of *F*. *fujikuroi* strains B14 or B20 were created using a split-marker recombination procedure as previously described [88]. To delete *FUM1*, the 5' and 3' flanking regions of the *FUM1* ORF were amplified with the primer pairs JFUM1f5/JFUM1rt5 and JFUM1ft3/JFUM1r3, respectively (in the first round of PCR), fused to the hygromycin B resistance gene (*hygB*) cassette, which was amplified from pBCATPH [88] with the primers HygB-for and HygB-rev (in the second round of PCR), and used as a template to generate split markers with the new nested primer sets, JFUM1fn/pUH-BC/H3 and JFUM1rn/pUH-BC/H2, respectively (in the third round of PCR) (S6 Table). Similarly, DNA constructs for targeted deletions of the other genes were created using the strategy described above. For the deletion of *FUB1*, the primer pairs JFUMB1f5/JFUM1Brt5 and JFUB1ft3/JFUB1r3 were used for amplification of 5' and 3' flanking regions of the *FUB1* ORF, respectively, and JFUB1fn/pUH-BC/H3 and JFUB1rn/pUH-BC/H2 were used as the nested primer sets, respectively. For the deletion of *PKS51*, the primer pairs B14_6372For5/B14_6372rev5t and B14_6372for3t/B14_6372rev3 were used in the first round of PCR, and B14_6372forN/ pUH-BC/H3 and B14_6372revN/ pUH-BC/H2 for the third round of PCR. Additionally, for double deletion of *FUM1* and *FUB1*, we generated a knock-out construct through which the 5′- and 3′-flanking regions of the *FUM1* ORF were fused to a geneticin resistance gene cassette (*gen*) amplified from pII99 using the primer pair Gen-for and Gen-rev, as described above. The resulting construct was transformed into the deletion strain of *FUB1*.

For the complementation of each deletion mutant of *FUM1* or *FUB1* derived from B14, intact copies of each gene were amplified from the genome of B14 using the primers JFUMf5/JFUMr3 and JFUB1f5/JFUBr3, respectively, and directly added into protoplasts of each deletion strain along with pSK660 including the geneticin resistance (*gen*) gene for co-transformation as previously described [89]. All primer sequences are listed in S6 Table.

### Pathotests on rice

The effect of the single isolates on rice seedling germination was studied by infecting the seeds with conidia of each isolate. For spore formation, the fungal strains were cultured on PDA plates for 7 days at 28°C under light/dark (12 h/12 h) conditions. The plates were flooded with sterile water to obtain a conidial suspension (1 x 10^−6^). Seeds were soaked in the suspension for 18 h. Inoculated and non-inoculated (control) seeds were sown into 100 mL plastic pots. Eight days after sowing the number of germinated seeds was assessed. The number of dead, chlorotic and elongated seedlings was measured 15 days after sowing.

For pathogenicity assays, healthy rice seeds were surface-sterilized by submersion in 70% of ethanol followed by 1% of sodium hypochlorite. Sterilized seeds were germinated in Murashige and Skoog (MS) agar [7] at 26°C for 5 days. The *Fusarium* isolates were first grown on oatmeal agar for one week. The pathogenicity assays were performed as previously described [7]. Agar plugs from the oatmeal plates were placed on top of 3 cm of sterilized vermiculite in a glass tube (18 cm x 1.6 cm). The agar plugs were then covered with 3 cm of vermiculite. Five-day-old seedlings were transferred to the surface of the vermiculite layer to avoid the direct contact between seedlings and fungal inocula. Before covering the tubes with a cap, 4 mL of Yoshida solution was gently added to each test tube to help retain high humidity [90] and placed at 26°C for 3, 5, 7 or 9 days. Their heights and internode lengths were measured and photographs of the seedlings and infected roots were taken. The symptom development caused by wild-type and mutant strains was examined in five independent pathogenicity tests. For pathotests with exogenous supply of culture filtrates, the wild-type B14 strain or its mutant lacking *FUM1* and *FUB1* (Δ*fum1*/Δ*fub1*) was inoculated into 50 mL of PDB (potato dextrose broth) and incubated for 5 days. The fungal liquid cultures were filtered through 2 × cheesecloth followed by filtration through 0.25 μm membranes. The culture fluid was dried to 5 ml by lyophilization (10-fold concentration). For inoculation assay, 500 μl of the concentrated culture filtrate was exogenously supplied to a single rice seedling.

### Transformations

*F*. *fujikuroi* B14 and B20 strains were transformed as previously described for *F*. *graminearum* [91]. Vector integration events were confirmed by diagnostic PCR (S10 Fig) using specific primers as indicated (S6 Table).

### PCR

PCR mixtures contained 25 ng of template DNA, 50 ng of each primer (S6 Table), 0.2 mM deoxynucleoside triphosphates, and 1 U of Biotherm Taq polymerase (Genecraft, Lüdinghausen, Germany). The cDNA synthesis was performed using Superscript II (Invitrogen, Groningen, The Netherlands) and 1.5 μg of total RNA as the template, according to the manufacturer's instructions. The qPCR was performed using iTaq Universal SYBR Green Supermix (BioRad) and Superscript II cDNA as template, in a Biorad thermocycler iTaq. In all cases, the qPCR efficiency was between 90–110% and the annealing temperature was 60°C. Gene expression was measured in three biological replicates from each time point, and the relative expression levels were calculated using the ΔΔCt method [92]. The expression of a translation elongation factor α gene (*EF1A*), amplified by a primer pair (EF1-PS1 and EF1-PS2) (S6 Table), was used as an endogenous reference for data normalization.

### Chemical analysis of secondary metabolites *in vitro* and *in planta*

For analyses of the SMs, the strains were grown in submerged cultures as described above. After 7 days, mycelia were removed from the culture by filtration through Mirachloth (Calbiochem, Merck KGaA, Darmstadt, Germany). The culture filtrates were filter-sterilized using 0.45 μm syringe filters (BGB, Schloßböckelheim, Germany).

Fusaric acid and beauvericin were obtained from Sigma-Aldrich (Deisenhofen, Germany), GAs from Serva (Heidelberg, Germany) and methylparaben (MePa) was obtained from Fluka (Steinheim, Neu-Ulm, Germany) in analytical grade. The remaining standard substances were obtained as described in previous work [24,25,34,35,42,93–95]. All solvents were obtained in gradient or analytical grade from Sigma-Aldrich, VWR (Darmstadt, Germany) or Merck Schuchardt (Hohenbrunn, Germany). Water was purified by a Milli-Q Gradient A 10 system (Millipore, Schwalbach, Germany).

Liquid culture samples were prepared as following: 10 μL of the culture filtrate and 10 μL of MePa (100 μg/mL) as internal standard were added to 80 μL of water. For *in planta* analysis, ten rice samples were combined and freeze-dried. The dried samples were treated with liquid nitrogen and pestled simultaneously, larger pieces were cut first with a scalpel. The samples were weighed and extracted with 1.5 mL of the following mixture: ethyl acetate:methanol (MeOH):dichloromethane, 3:2:1. Precellys ceramic beads 1.4/2.8 (Peqlab, Erlangen, Germany) were added to the samples, and the mixture was vortexed for 1 min. Afterwards, the samples were shaken for 1 h on a rotary shaker with 150 rpm. After a short centrifugation step (3 min, 2900 g), 500 μL of the supernatant were transferred to a new vial and the solvent was evaporated to dryness under constant nitrogen flow. The residue was dissolved in 100 μL MeOH and put to an ultrasonic bath for 10 min. After vortexing the samples, they were centrifuged again with 5000 g, and 50 μL of the supernatant were collected. Afterwards, the samples were dried again under nitrogen flow and 1.5 mL of MeOH/water, 3/1 (v/v), + 0.1% formic acid (FA) were added. The extraction procedure described above was repeated. 10 μL of each extract were mixed with 10 μL MePa and 80 μL water for analysis. Some extracts were diluted again 1:10; the corresponding values are labelled.

The liquid culture samples were analyzed as following: A Shimadzu LC-20AD HPLC (Shimadzu, Kyoto, Japan) with a SIL-20ACXR autosampler coupled to a Sciex QTRAP 5500 (Sciex, Darmstadt, Germany) mass spectrometer was used. Separation was carried out on a Synergi Hydro-RP column from Phenomenex (Aschaffenburg, Germany) with 50 × 2.0 mm and 2.5 μm particle size, protected by a KrudKatcher classic filter (Phenomenex), and on a Nucleodur C18 Gravity-SB column from Macherey-Nagel (Düren, Germany) with 50 × 2.0 mm and 3 μm particle size, protected by a KrudKatcher classic filter (Phenomenex), at 50°C. MeOH + 1% FA + 5 mM NH_4_Ac was used as eluent A, water + 1% FA + 5 mM NH_4_Ac was used as eluent B. A flow volume of 450 μL/min was applied, and the gradient started at 5% A. This condition was held for 1.5 min. The gradient rose up to 98% A in 10.5 min, and finally the column was rinsed for 3 min with 98%. After that, the column was equilibrated for 2.5 min with 5% A. The integrated valco valve was used, discarding the first 2 min of the run, and the samples were cooled to 7°C. 5 μL of each sample was injected.

Advanced scheduled multiple reaction monitoring (MRM) mode was used for acquisition. Both positive and negative ionization mode were applied. The curtain gas (CUR) was set to 35 psi, the collision gas was set to medium. The temperature of the heater gas (TEM) in the ion source was set to 450°C. Nebulizer gas (GS1) and auxiliary gas (GS2) were adjusted to 35 and 45 psi, respectively.

In the positive MRM mode, the ion spray voltage (IS) was set to 4500 V, and the target scan time of this experiment was adjusted to 0.3 s, resulting in a cycle time of 0.5 s. The positive ionization mode was applied for the relative quantification of *O*-methyl-fusarubin, fusaric acid, gibepyrone A, apicidin F, beauvericin, fumonisins (FB_1_+FB_2_), fusarins, trichosetin and fujikurin A. The cell exit potential (CXP) was set to 11 V, the entrance potential (EP) was set to 10 V. For the negative MRM mode, the IS was set to -4500 V and the target scan time was adjusted to 0.2 s, resulting in a cycle time of 0.5 s. The negative ionization mode was applied for the relative quantification of MePa, and the gibberellins GA_3_, GA_4_ and GA_7_. The CXP was set to 11 V, the EP was set to 10 V.

MRM transition for the quantification were as follows: FB_1_−722.4–223.1; FB_2_−706.4–336.3; GA_3_−345.2–143.0; GA_4_−331.3–243.1; GA_7_−331.3–243.1. The calibration curve for all the standards was prepared in a concentration range of 0.0001–1 μg/ml.

For bikaverin determination in liquid culture samples, HPLC-UV measurements were carried out on a Shimadzu LC-20AT pump system with a Shimadzu SIL autosampler and a photodiode array (PDA). A Gemini 5 u C6-Phenyl 110A, 250 × 4.60 mm, 5 μm column (Phenomenex) was used, with water + 1% FA as eluent A and acetonitril + 1% FA as eluent B. The column oven was set to 40°C. The gradient started with 10% B with 1.35 μL/min. After 3 min, the gradient rose up to 100% B during 17 min. The column was washed with 100% B for 6 min, and afterwards the column was equilibrated with 10% B again for 4 min. The wavelength for PDA analysis ranged from 220–600 nm. 100 μL of the sterile culture filtrate were injected. Peak areas were determined at 508 nm, and bikaverin obtained from Sigma Aldrich was used as standard substance.

*In planta* sample analysis was performed with a different HPLC-system but the same mass spectrum. An Agilent 1260 HPLC system (Santa Clara, USA) was used, and the expected retention time for scheduled MRM analysis needed to be adjusted (S7–S10 Tables). Furthermore, bikaverin was analyzed in positive ionization mode.

## Supporting information

S1 Fig PCR validation of telomere-proximal genes in all strains according to reference sequence of strain IMI 58289 (Primer list see S6 Table). (TIF)

S2 Fig Northern blot analysis for comparison of bikaverin (*BIK2*) and fusarubins (*FSR2*) gene expression. The strains were grown for 3 days in synthetic medium with either 6 mM glutamine (bikaverin) or 6 mM NaNO_*3*_ (fusarubins) as nitrogen source.
(TIF)

S3 Fig Content and arrangement of genes in gene clusters with variations in the single isolates. (A) The apicidin F (NRPS31) gene cluster is present in most isolates but missing in strain B14. (B) The fumonisin (PKS11) gene cluster is present in most of the strains, but several genes of the cluster are missing in strain B20. Arrows in blue represent genes belonging to a specific gene cluster. Green arrows represent genes that have closely related homologs in two or more isolates while light-gray arrows represent genes that do not have closely related homologs in other isolates. Ψ indicates a pseudogene.
(TIF)

S4 Fig Rice seedlings inoculated with *F*. *fujikuroi* wild-type and mutant strains with or without addition of culture fluids to the pathosystem. (A) Shoot growth of rice seedlings inoculated with B14 (wild type) and the B14 deletion and complemented strains 9 days after inoculation. (B, C, D) Shoot growth of rice seedlings inoculated with strain B14 (B), B20 (C) or the GA-deficient mutant strain of B20 (B20 Δ*cps*/*ks*) (D) with or without the exogenous supply of the culture filtrate of B14 or its Δ*fum1*/Δ*fub1* mutant. Mock: no fungal inoculation; Δ*fum1* and Δ*fub1 –*deletion strains for the fumonisin and fusaric acid key genes, respectively; *FUM1*^*C*^ and *FUB1*^*C*^ –complemented strains carrying an intact copy of *FUM1* or *FUB1*, respectively.
(TIF)

S5 Fig Shoot and root growth of rice seedlings after inoculation with B20 mutants. Rice seedlings (A-C) and roots (D) inoculated with the gene deletion strains derived from the *F*. *fujikuroi* B20 strain. Mock: no fungal inoculation; Δ*fum1*, Δ*fub1*, and Δ*cps/ks–*deletion strains for the fumonisin, fusaric acid and giberellic acid key genes, respectively.
(TIF)

S6 Fig **Shoot (A) and root (B) growth of rice seedlings 7 days after inoculation of high fumonisin-producing *F*. *verticillioides* strains.** Mock: no fungal inoculation; B14: the *F*. *fujikuroi* B14 strain; FvOS35: the *F*. *verticilliodies* OS35 strain; FvOS40: the *F*. *verticilliodies* OS40 strain.
(TIF)

S7 Fig Differentiation of field isolates for their B14-like or B20-like pathotype by PCR and phylogenetic analysis. (A) Diagnostic PCR amplification using the primer pairs derived from *PKS51* (unknown product, 382 bp) and *NRPS31* (apicidin F, 434 bp), respectively. (B) Phylogenetic tree constructed by the NJ method using the nucleotide sequences of combined *TEF1* and *RPB2* from additional field isolates, determined by the diagnostic PCR of (A).
(TIF)

S8 Fig Relative transcript levels for the key genes of GA (*CPS*/*KS*) and fumonisin (*FUM1*) biosynthesis. The transcript levels of *CPS*/*KS* (A) and *FUM1* (B) were determined by qPCR using total RNA from several *F*. *fujikuroi* field isolates grown in ICI liquid medium containing 6 mM glutamine for 7 d. Amplification levels of *CPS*/*KS* and *FUM1* in the B14 strain were used as a reference (set to 1.0). The same letter above bars indicates no significant difference.
(TIF)

S9 Fig **Biosynthesis of gibberellic acids (GA**_**3**_**, GA**_**4**_**, GA**_**7**_**) and fumonisins (FB**_**1**_**, FB**_**2**_**) of stunting-type *F*. *fujikuroi* isolates compared to *bakanae* strains under *in vitro* (A, B) and *in planta* (C, D) conditions.** GA (A) and fumonisin (B) production levels after 7 days of growth in synthetic medium with 6 mM glutamine. The strains were grown in triplicates. GA (C) and fumonisin (D) production levels in rice roots and shoots 7 dpi. For *in planta* analyses, ten plants per isolate were used.
(TIF)

S10 Fig **Confirmation of the gene deletions (A-F) or complementations (G and H) by PCR.** Left panel in each figure: gene deletion or complementation schemes, right panel: PCR gel picture. The genomic positions of the primer pairs (S6 Table) used in the PCR amplification and expected size of PCR products (designated a or b) are indicated in the deletion schemes and the gels, respectively. In most cases, two independent strains with a gene deletion or a gene add-back (designated 1 and 2) were used in PCR along with their wild-type (WT) progenitor and those carrying a single gene deletion (SD). For gene complementation, we used a co-transformation strategy as previously described [88].
(TIF)

S1 Table BUSCO single-copy analysis, performed in gene set (protein) assessment mode on the library Sordariomyceta_odb9. (DOCX)

S2 Table Distribution of the mating type loci among the *F*. *fujikuroi* isolates. (XLSX)

S3 Table Presence of secondary metabolite gene clusters in the analyzed *Fusarium* strains. (XLSX)

S4 Table Rice seed germination assay. (DOCX)

S5 Table (A) Expression data and list of all genes with a transcription factor specific InterPro ID in the corresponding protein sequence. (B) Expression data and list of 37 genes which are present in most of the 9 genomes and which are specifically up-regulated during infection of rice. (C) Expression data and list of 28 B14 strain-specific TFs which are not present in the genomes of the other strains.
(XLSX)

S6 Table PCR primers used in this work. (DOCX)

S7 Table MRM transitions for liquid culture analysis in positive ionization mode, depicting also transition specific variables. (DOCX)

S8 Table MRM transitions for liquid culture analysis in negative ionization mode, depicting also transition specific variables. (DOCX)

S9 Table MRM transitions for *in planta* analysis in positive ionization mode, depicting also transition specific variables. (DOCX)

S10 Table MRM transitions for *in planta* analysis in negative ionization mode, depicting also transition specific variables. (DOCX)
