# Supplementary figures and images for: Comparative genomics of geographically distant Fusarium fujikuroi isolates revealed two distinct pathotypes correlating with secondary metabolite profiles
Source: PLoS Pathog. 2017 Oct 26;13(10):e1006670. doi: 10.1371/journal.ppat.1006670 (PMC5675463; doi:10.1371/journal.ppat.1006670)

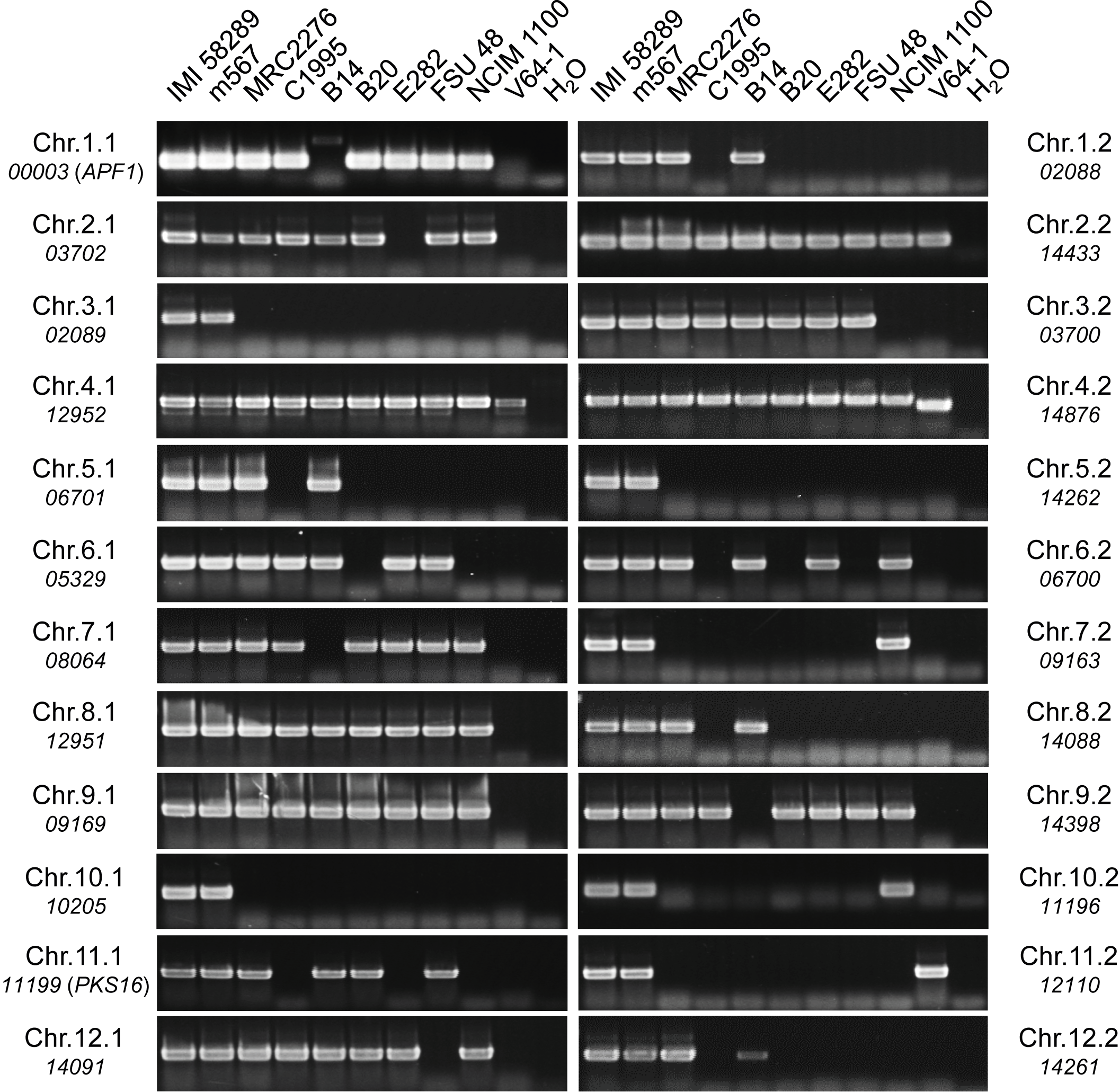

Supplement: S1 Fig — (TIF) [file ppat.1006670.s001.tif]

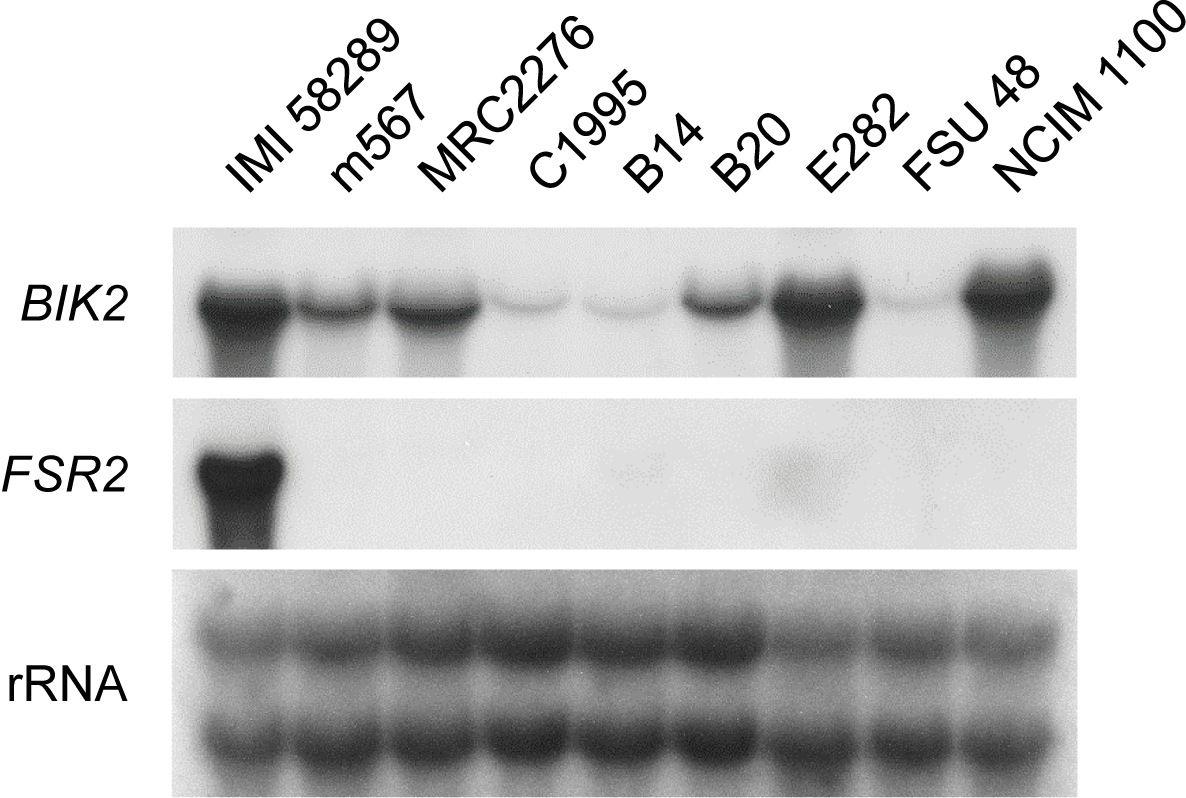

Supplement: S2 Fig — The strains were grown for 3 days in synthetic medium with either 6 mM glutamine (bikaverin) or 6 mM NaNO3 (fusarubins) as nitrogen source. (TIF) [file ppat.1006670.s002.tif]

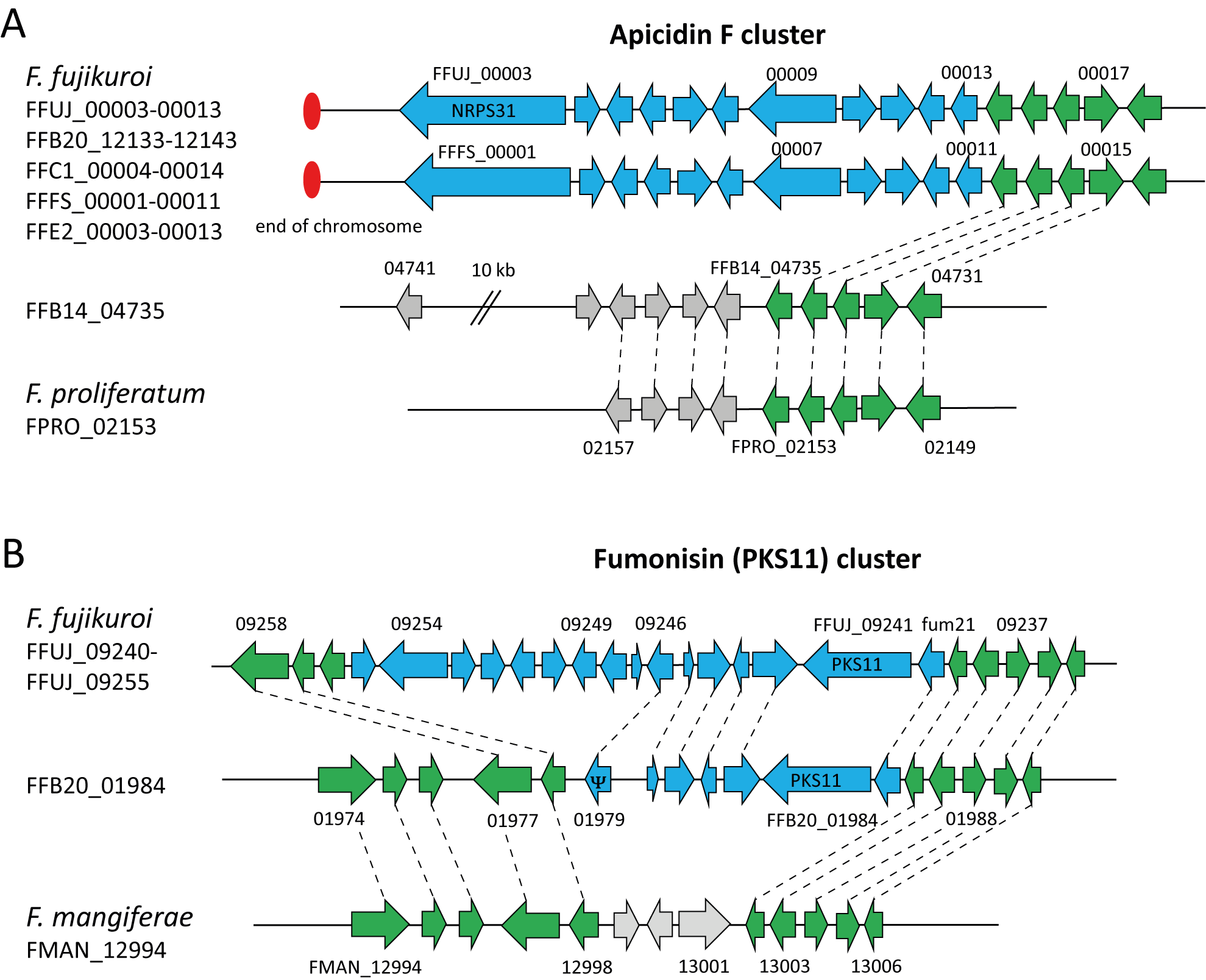

Supplement: S3 Fig — (A) The apicidin F (NRPS31) gene cluster is present in most isolates but missing in strain B14. (B) The fumonisin (PKS11) gene cluster is present in most of the strains, but several genes of the cluster are missing in strain B20. Arrows in blue represent genes belonging to a specific gene cluster. Green arrows represent genes that have closely related homologs in two or more isolates while light-gray arrows represent genes that do not have closely related homologs in other isolates. Ψ indicates a pseudogene. (TIF) [file ppat.1006670.s003.tif]

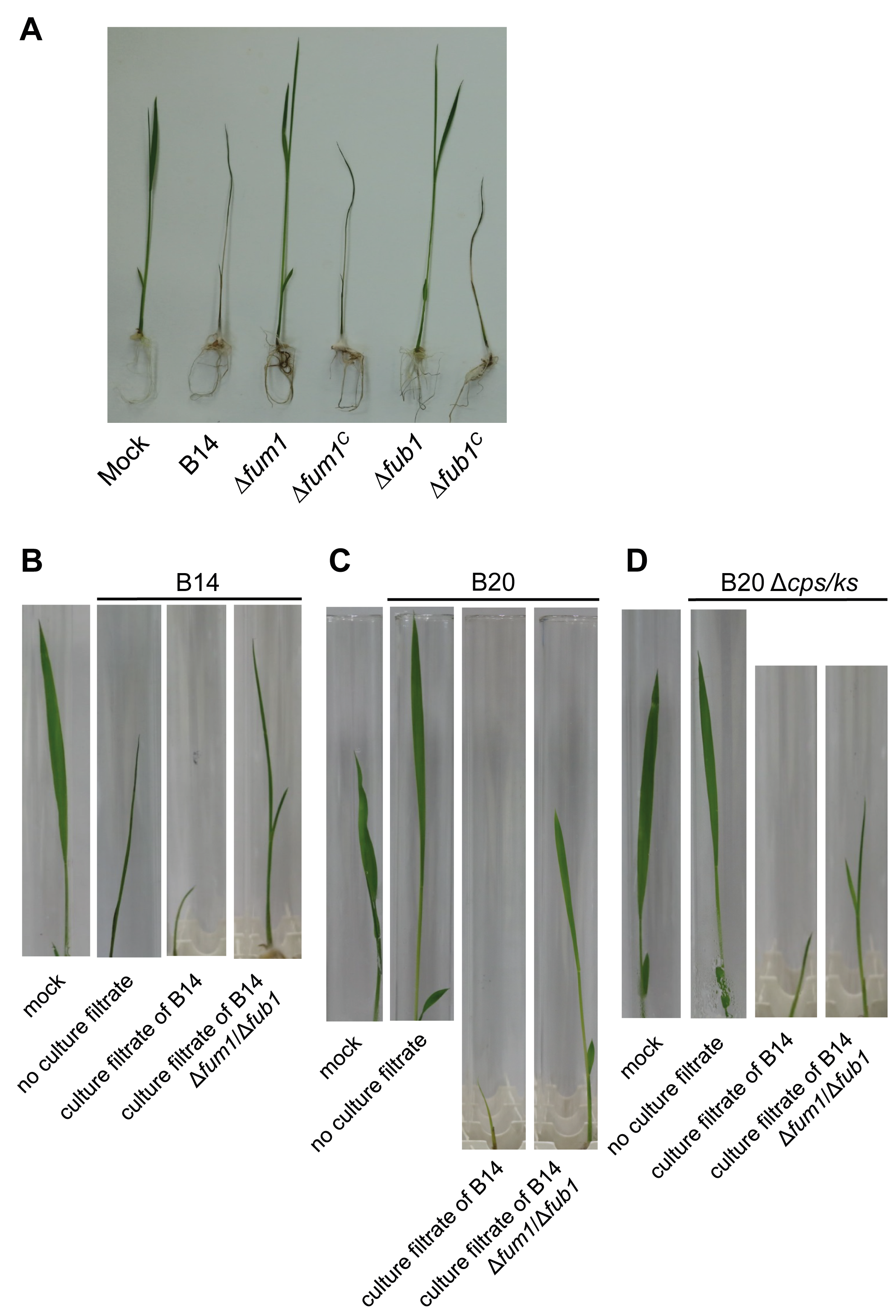

Supplement: S4 Fig — (A) Shoot growth of rice seedlings inoculated with B14 (wild type) and the B14 deletion and complemented strains 9 days after inoculation. (B, C, D) Shoot growth of rice seedlings inoculated with strain B14 (B), B20 (C) or the GA-deficient mutant strain of B20 (B20 Δcps/ks) (D) with or without the exogenous supply of the culture filtrate of B14 or its Δfum1/Δfub1 mutant. Mock: no fungal inoculation; Δfum1 and Δfub1 –deletion strains for the fumonisin and fusaric acid key genes, respectively; FUM1C and FUB1C –complemented strains carrying an intact copy of FUM1 or FUB1, respectively. (TIF) [file ppat.1006670.s004.tif]

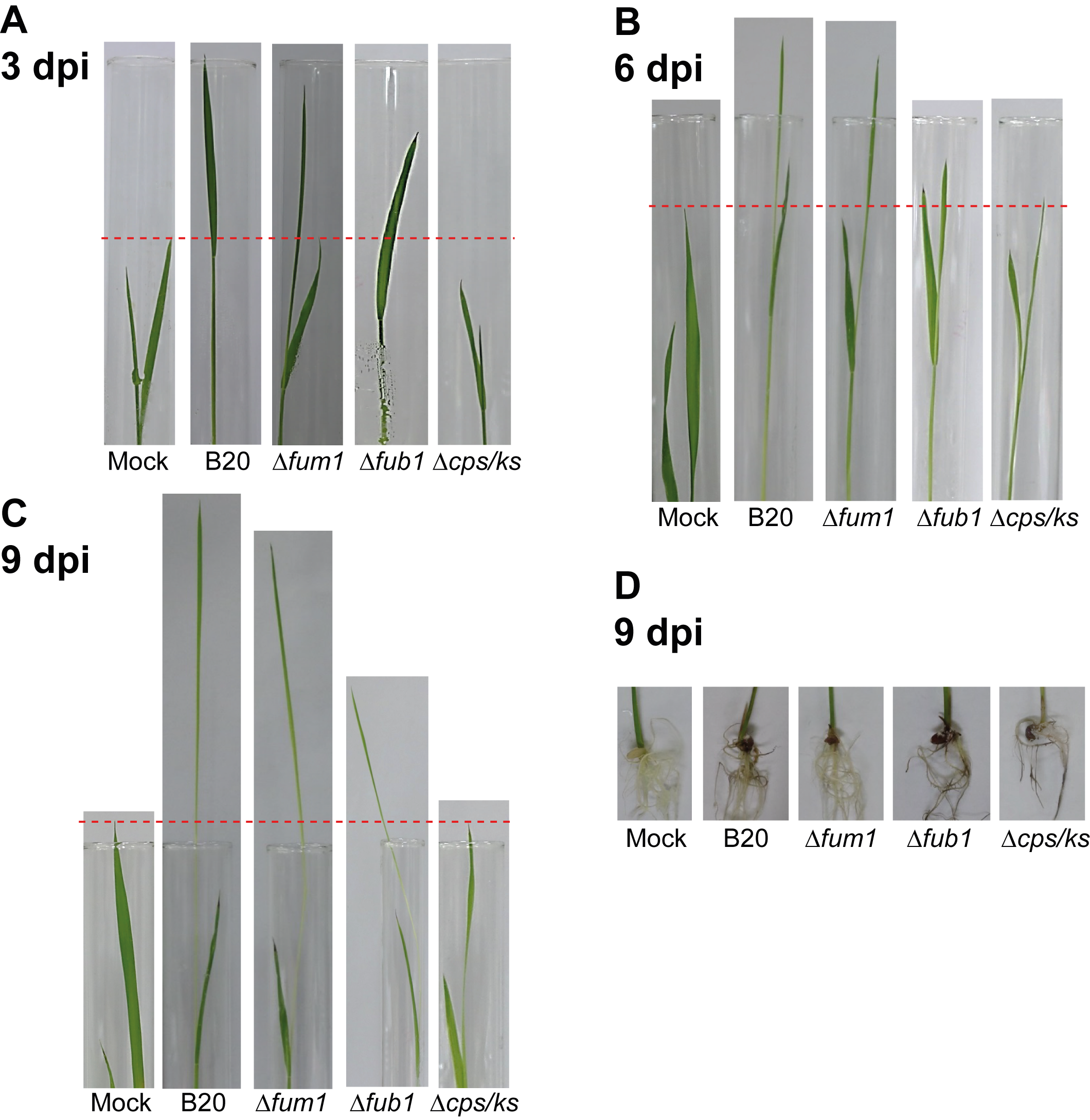

Supplement: S5 Fig — Rice seedlings (A-C) and roots (D) inoculated with the gene deletion strains derived from the F. fujikuroi B20 strain. Mock: no fungal inoculation; Δfum1, Δfub1, and Δcps/ks–deletion strains for the fumonisin, fusaric acid and giberellic acid key genes, respectively. (TIF) [file ppat.1006670.s005.tif]

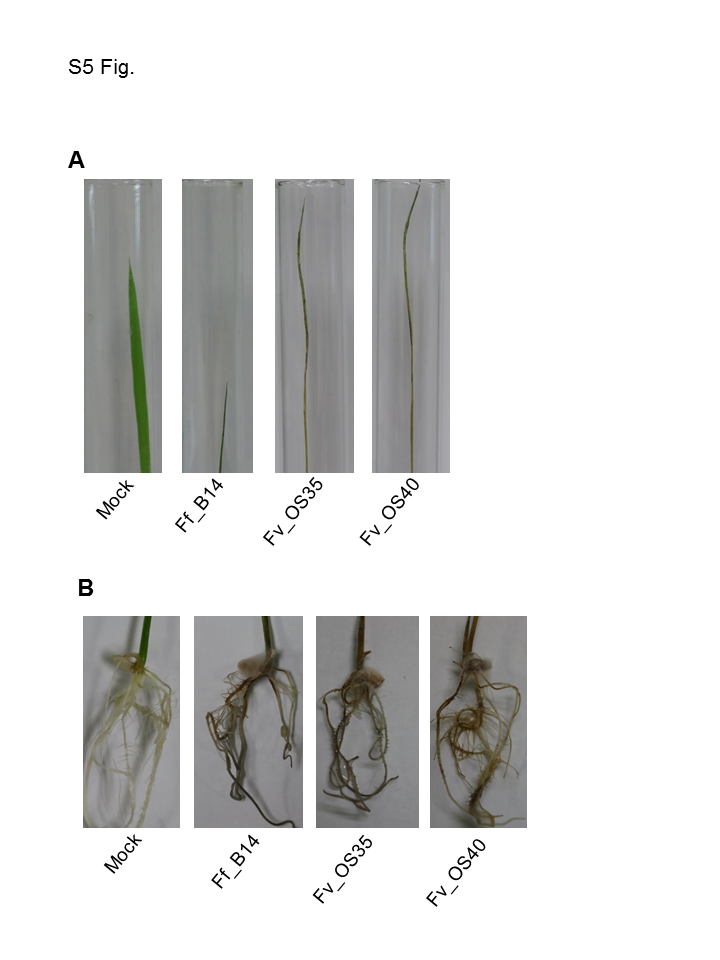

Supplement: S6 Fig — Shoot (A) and root (B) growth of rice seedlings 7 days after inoculation of high fumonisin-producing F. verticillioides strains. Mock: no fungal inoculation; B14: the F. fujikuroi B14 strain; FvOS35: the F. verticilliodies OS35 strain; FvOS40: the F. verticilliodies OS40 strain. (TIF) [file ppat.1006670.s006.tif]

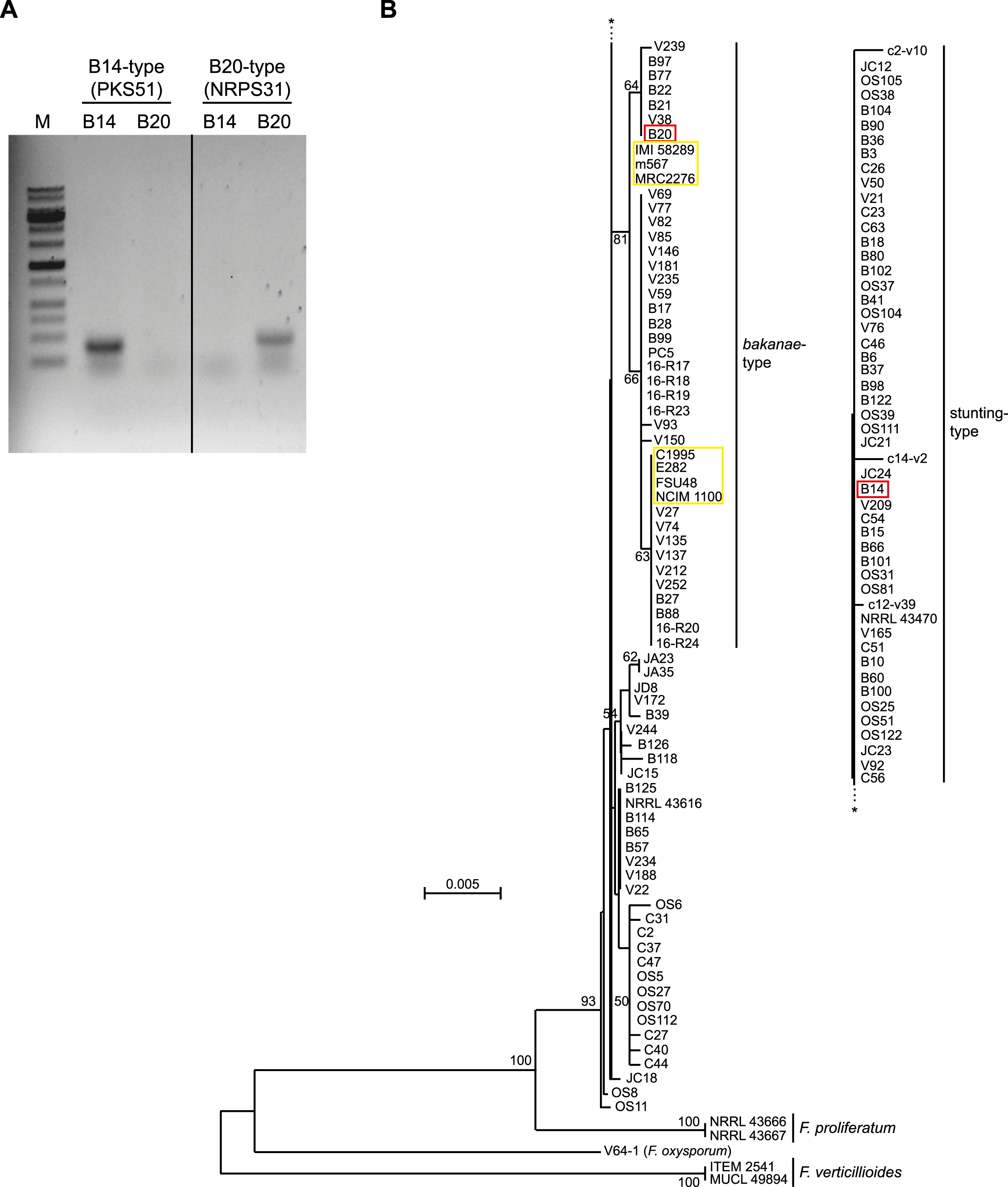

Supplement: S7 Fig — (A) Diagnostic PCR amplification using the primer pairs derived from PKS51 (unknown product, 382 bp) and NRPS31 (apicidin F, 434 bp), respectively. (B) Phylogenetic tree constructed by the NJ method using the nucleotide sequences of combined TEF1 and RPB2 from additional field isolates, determined by the diagnostic PCR of (A). (TIF) [file ppat.1006670.s007.tif]

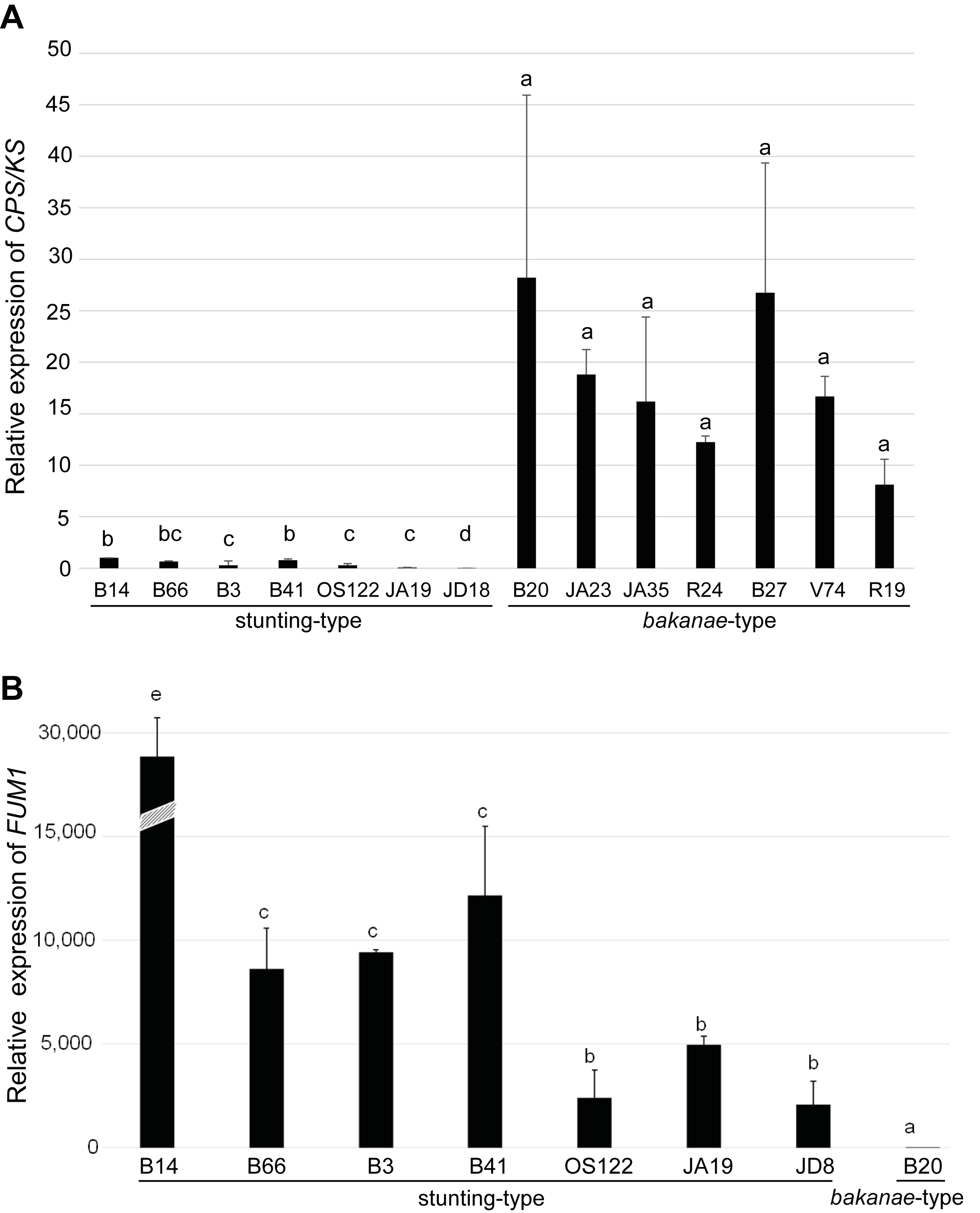

Supplement: S8 Fig — The transcript levels of CPS/KS (A) and FUM1 (B) were determined by qPCR using total RNA from several F. fujikuroi field isolates grown in ICI liquid medium containing 6 mM glutamine for 7 d. Amplification levels of CPS/KS and FUM1 in the B14 strain were used as a reference (set to 1.0). The same letter above bars indicates no significant difference. (TIF) [file ppat.1006670.s008.tif]

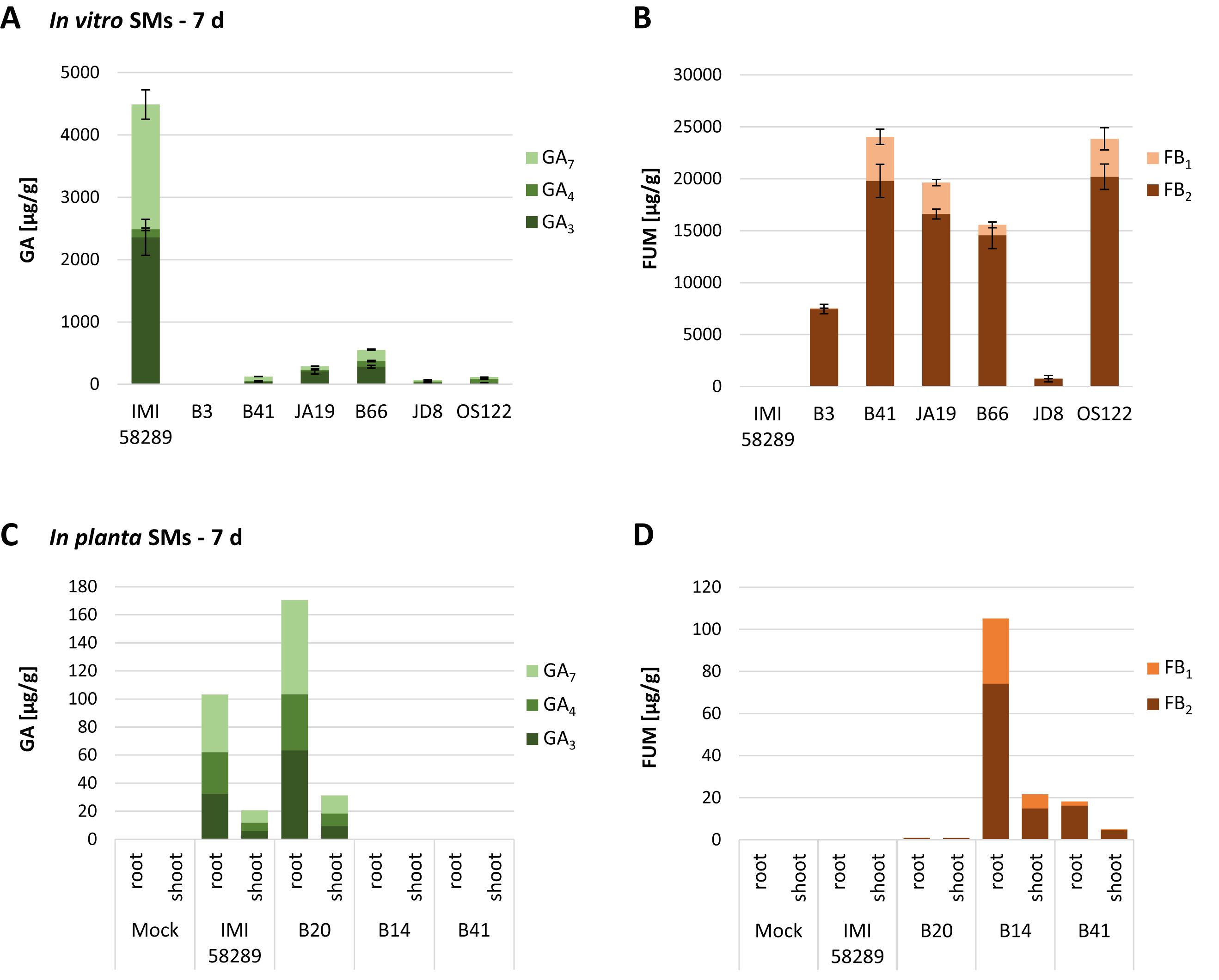

Supplement: S9 Fig — Biosynthesis of gibberellic acids (GA3, GA4, GA7) and fumonisins (FB1, FB2) of stunting-type F. fujikuroi isolates compared to bakanae strains under in vitro (A, B) and in planta (C, D) conditions. GA (A) and fumonisin (B) production levels after 7 days of growth in synthetic medium with 6 mM glutamine. The strains were grown in triplicates. GA (C) and fumonisin (D) production levels in rice roots and shoots 7 dpi. For in planta analyses, ten plants per isolate were used. (TIF) [file ppat.1006670.s009.tif]

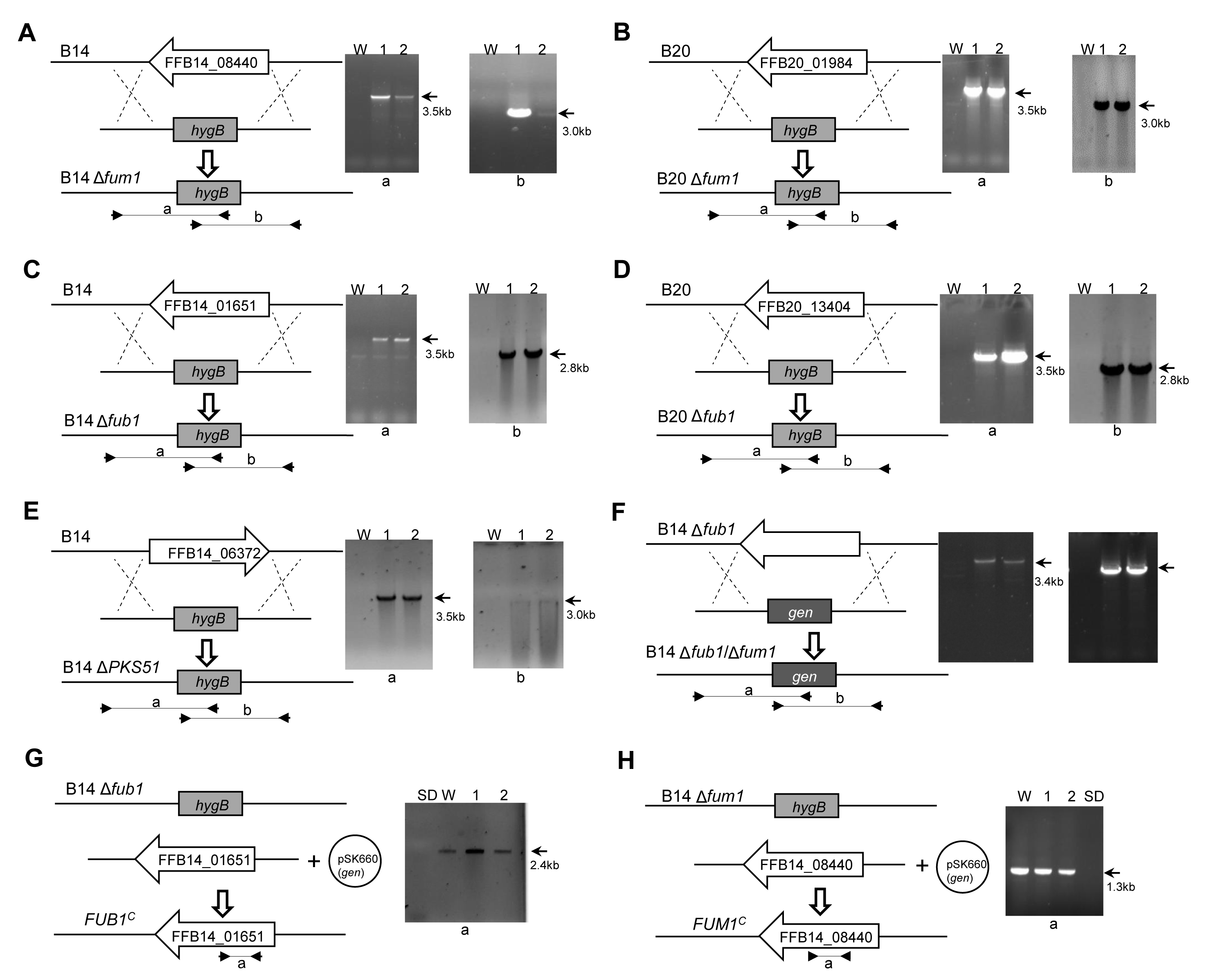

Supplement: S10 Fig — Confirmation of the gene deletions (A-F) or complementations (G and H) by PCR. Left panel in each figure: gene deletion or complementation schemes, right panel: PCR gel picture. The genomic positions of the primer pairs (S6 Table) used in the PCR amplification and expected size of PCR products (designated a or b) are indicated in the deletion schemes and the gels, respectively. In most cases, two independent strains with a gene deletion or a gene add-back (designated 1 and 2) were used in PCR along with their wild-type (WT) progenitor and those carrying a single gene deletion (SD). For gene complementation, we used a co-transformation strategy as previously described [88]. (TIF) [file ppat.1006670.s010.tif]
