# Supplementary material for: Comparative genomics of geographically distant Fusarium fujikuroi isolates revealed two distinct pathotypes correlating with secondary metabolite profiles
Source: PLoS Pathog. 2017 Oct 26;13(10):e1006670. doi: 10.1371/journal.ppat.1006670 (PMC5675463; doi:10.1371/journal.ppat.1006670)
Supplement: S1 Table — (DOCX) [file ppat.1006670.s011.docx]

**S1 Table. BUSCO single-copy analysis, performed in gene set (protein) assessment mode on the library Sordariomyceta_odb9.**

| **Species** | **Strain** | **BUSCO notation assessment results** |
| --- | --- | --- |
| *F. fujikuroi* | B14 | C:99.1%[S:98.9%, D:0.2%], F:0.7%, M:0.2%, n:3725 |
| *F. fujikuroi* | B20 | C:98.4%[S:97.9%, D:0.5%], F:0.9%, M:0.7%, n:3725 |
| *F. fujikuroi* | C1995 | C:99.2%[S:98.9%, D:0.3%], F:0.7%, M:0.1%, n:3725 |
| *F. fujikuroi* | E282 | C:99.2%[S:99.0%, D:0.2%], F:0.6%, M:0.2%, n:3725 |
| *F. fujikuroi* | FSU48 | C:99.1%[S:98.8%, D:0.3%], F:0.7%, M:0.2%, n:3725 |
| *F. fujikuroi* | IMI58289 | C:99.1%[S:98.8%, D:0.3%], F:0.9%, M:0.0%, n:3725 |
| *F. fujikuroi* | m567 | C:99.2%[S:99.0%, D:0.2%], F:0.7%, M:0.1%, n:3725 |
| *F. fujikuroi* | MRC2276 | C:99.1%[S:98.9%, D:0.2%], F:0.7%, M:0.2%, n:3725 |
| *F. fujikuroi* | NCIM1100 | C:99.1%[S:98.9%, D:0.2%], F:0.8%, M:0.1%, n:3725 |
| *F. oxysporum* | V64-1 | C:99.0%[S:98.4%, D:0.6%], F:0.8%, M:0.2%, n:3725 |
| *F. oxysporum* | 4287 | C:94.2%[S:69.6%, D:24.6%], F:3.7%, M:2.1%, n:3725 |
| *F. proliferatum* | ET1 | C:99.3%[S:98.9%, D:0.4%], F:0.6%, M:0.1%, n:3725 |
| *F. proliferatum* | NRRL62905 | C:99.0%[S:98.7%, D:0.3%], F:0.8%, M:0.2%, n:3725 |
| *F. mangiferae* | MRC7560 | C:99.1%[S:98.7%, D:0.4%], F:0.7%, M:0.2%, n:3725 |
| *F. verticillioides* | 7600 | C:97.6%[S:74.4%, D:23.2%], F:1.6%, M:0.8%, n:3725 |
| *F. graminearum* | PH1 version 3.2 | C:94.7%[S:94.5%, D:0.2%], F:3.9%, M:1.4%, n:3725 |

C: Complete; S: Single-copy; D: Duplicated; F: Fragmented; M: Missing
