# Supplementary material for: Comparative genomics of geographically distant Fusarium fujikuroi isolates revealed two distinct pathotypes correlating with secondary metabolite profiles
Source: PLoS Pathog. 2017 Oct 26;13(10):e1006670. doi: 10.1371/journal.ppat.1006670 (PMC5675463; doi:10.1371/journal.ppat.1006670)
Supplement: S4 Table — (DOCX) [file ppat.1006670.s014.docx]

**Table S4: Rice seed germination assay**

| **Strain number** | **% germinated seeds (14 days)*** | **% living seedlings developed from germinated seeds**  **(14 days)** |
| --- | --- | --- |
| **1. IMI 58289** | 84.4 ab | 24 |
| **2. m567** | 100.0 a | 63.6 |
| **3. MRC2276** | 71.9 abc | 100 |
| **4. C1995** | 31.2 bcd | 100 |
| **5. B14** | 18.7 cd | 100 |
| **6. B20** | 0.0 d | no seedlings alive |
| **7. E282** | 50.0 abcd | 95.2 |
| **8. FSU 48** | 50.0 abcd | 100 |
| **9. NCIM 1100** | 96.9 a | 17.4 |
| **10. V64-1** | 96.9 a | no symptoms |
| **11. not inoculated** | 100 a | no symptoms |

*Means were statistically compared using the Tukeys HSD Test (P≤0.05). Values followed by the same letters were not significantly different from each other.
