# Supplementary material for: Comparative genomics of geographically distant Fusarium fujikuroi isolates revealed two distinct pathotypes correlating with secondary metabolite profiles
Source: PLoS Pathog. 2017 Oct 26;13(10):e1006670. doi: 10.1371/journal.ppat.1006670 (PMC5675463; doi:10.1371/journal.ppat.1006670)
Supplement: S6 Table — (DOCX) [file ppat.1006670.s016.docx]

**Table S6. PCR primers used in this work.**

| **Primer** | **Sequence 5‘ 🡪 3‘** |
| --- | --- |
| **Primers for chromosome ends** | |
| Chr.1.1_00003_F  Chr.1.1_00003_R  Chr.1.2_02088_F  Chr.1.2_02088_R  Chr.2.1_03702_F  Chr.2.1_03702_R  Chr.2.2_14433_F  Chr.2.2_14433_R  Chr.3.1_02089_F  Chr.3.1_02089_R  Chr.3.2_03700_F  Chr.3.2_03700_R  Chr.4.1_12952_F  Chr.4.1_12952_R  Chr.4.2_14876_F  Chr.4.2_14876_R  Chr.5.1_06701_F  Chr.5.1_06701_R  Chr.5.2_14262_F  Chr.5.2_14262_R  Chr.6.1_05329_F  Chr.6.1_05329_R  Chr.6.2_06700_F  Chr.6.2_06700_R  Chr.7.1_08064_F  Chr.7.1_08064_R  Chr.7.2_09163_F  Chr.7.2_09163_R  Chr.8.1_12951_F  Chr.8.1_12951_R  Chr.8.2_14088_F  Chr.8.2_14088_R  Chr.9.1_09169_F  Chr.9.1_09169_R  Chr.9.2_14398_F  Chr.9.2_14398_R  Chr.10.1_10205_F  Chr.10.1_10205_R  Chr.10.2_11196_F  Chr.10.2_11196_R  Chr.11.1_11199_F  Chr.11.1_11199_R  Chr.11.2_12110_F  Chr.11.2_12110_R  Chr.12.1_14091_F  Chr.12.1_14091_R  Chr.12.2_14261_F  Chr.12.2_14261_R | GGAGATACAATTGCCGG  GCGTCGATGGCGGTGTGTGACG  CTGCTAGCTGCCCGTGGGC  TGAAAGCCGGCAACTTCAACG  CCCTTGGCCACCTCTAGCCC  GAATCGCTGGGTCGCTGGC  CGATTTGCACGTCAGAACCGC  GTGCACTTTTCCAGTCCAGGCC  CCTTCCACAAGGTCACCCATCC  CATTGGGATGCAGGGTCTGCC  CGATGTCGGTTCTTCTCCCTCC  TGCACTCGATGCTGGGCACC  CGGCGCCGAGGTCAATGG  GCTCTTCTCGGTGCTGCCGG  CACTTCCACGATGTGGCTGGC  GGGCATGTGGTAGCACCAGTCC  GGAGGATCTATGGCAGTCGATTCC  GGGAAATAGCCTCGAGCTGCG  GGGAGCTCATCGGACAGTGGC  ACGCAAGGGATGCCATTAGGG  CCGCATGTCCATATTCTTCGCG  CAGGGTGCTTGCCATGATGACC  ATCATCCTTGGCTTCGGCGC  GGCGCGAGCTGAGAGGCC  CCCACAAGAAGCGTTGCCTACG  CGGTCGCTACTCCAGGCACC  CGAGAAACGCTGTGCTTCGAGG  GCAGACCCTCGTGTGGCACC  CAAGTGAGTGACGCGGCGC  CCCTCAATACGGGCTCCTCGC  CGTCATTGCTACAGTGTCTCTCAGC  TCAGTCGCCTCCATGACAATAGC  GCCCTCTAGCTCGCCTCCAGC  ACGAGGATGGCGAGGATGGG  CGGTATATGCAAGAGGCTCGGC  CAATCCTGGATTCCCGAGCTCC  CCGCCTGAGATTTCAGAGGCTG  CCCGTCGGGATTAGTGAGAAAGC  CGCATCATCAGCCAGATAACCG  TTGCTTAAGTGCAAAGATGCTAGCC  GGCGACGCAATTCCAGAGCC  CTGATTGCAGGCAGTGCCTCG  TCTACGCGCAACATGTTCGGC  CAAGCTGTTCGCTTGAGCCGC  CAACAACCGCCACAGGCACC  GGAGGAACAGTGTCATTCCCGC  CGCTTCAACAACGACCATCGG  CCCTTGCAGGCATATTAGAGCCG |
| **Primers for amplification of PKS51 and NRPS31 fragments** | |
| B14J06375F2 | AGAAAGATCGTGCGACTATGAAGA |
| B14J06375R2 | TTGGTGCGTACGACTGGGTGAAC |
| B20J12141F2 | TACGAGAGGGAAACCAAAGAACAC |
| B20J12141R2 | CATGTGAACGCAAGCGAGAAATAG |
| **Primers for probes for Northern blot** | |
| cps/ks-F  cps/ks-R  bik2-F  bik2-R  fsr2-F  fsr2-R  Fum8-F  Fum8-R | GTGTAGCTGGATCATAGCGACACTCCTG  CCATTGGCCCTGGCTAAGTTTCCC  CTTGAGTCTGATAGAGGCGC  ACGGCGCAGCAGAAAGTGCC  TGTCATTGAGCATGCTACGC  ATCAGCCTTGGTAAGCAGGG  AGTGGTGGCAAGATTGTGG  ATCGTCGAGGTATTGCTTCG |
| **Primers for generation of knock out vectors** | |
| JFUM1f5 | GTAGCTGTGAGGCGTTGGCGTAT |
| JFUM1rt5 | CAGGTACACTTGTTTAGCTGGCGAAGGTAAATAGAATGA |
| JFUM1ft3 | TCAATATCATCTTCTGTCGAGAACCGAGTAGACTATCCATTGC |
| JFUM1r3 | AGATTTCTTGAACTGACCGTATTAT |
| JFUM1fn | GCTGGGCCACGGGAAGCACTACA |
| JFUM1rn | CTGTTGGATGAATGGAGAGGTGAC |
| JFUB1f5 | TACCTTTTTACAATCTCGGCAACCTA |
| JFUB1rt5 | CAGGTACACTTGTTTAGTCAGTTCATTTCTTAGTTCGCTTAG |
| JFUB1ft3 | TCAATATCATCTTCTGTCGTTCGGAAGATATTGACCCTAAGAT |
| JFUB1r3 | TGAGCGGCAGAGACAGAGCACTAA |
| JFUB1fn | ATGTTCTCGGCAAGCCCAAAAATAGC |
| JFUB1rn | GCGAGGATCACGGCAGTCATAG |
| B14_6372For5 | AAGGACTTGAGATCGTGAGAG |
| B14_6372rev5t | CAGGTACACTTGTTTAGAGATTGGTTGTTGCTTAGTTTATTG |
| B14_6372for3t | TCAATATCATCTTCTGTCGGGGTCCAAGCTAGGTTATGA |
| B14_6372rev3 | AGCCCAATTCTCAAGTGTCA |
| B14_6372forN | ATGTCGTGAGGTAAAAGGGCAATGT |
| B14_6372revN | GTGGATATGATTAGCGCGAACAGAGT |
| HygB-for | CTCTAAACAAGTGTACCTGTGC |
| HygB-rev | CGACAGAAGATGATATTGAAGG |
| pUH-BC/H3 | GCTGCTCCATACAAGCCAACC |
| pUH-BC/H2 | CGTTATGTTTATCCTGCACTTTGC |
| Gen-forN (g3) | TGCTGCTTGGACAAATGAACG |
| Gen-revN (g2) | CTTTTCTGGATTCATCGACTGTGG |
| **Primers for qPCR** | |
| EF1-PS1 | GGCTTTCACCGACTACCCTCCTCT |
| EF1-PS2 | ACTTCTCGACGGCCTTGATGACAC |
| qcps.ksFor3 | GGCACCTTTCCTACAACACATTTT |
| qcps.ksRev3 | ATTACTCCATTCTCGTCCCTCAA |
| **Primers for diagnostic PCR to confirm gene replacement** | |
| qFUM-r3 (p11) | CTGCGCATACAAGTGGCTGAGA |
| qFUM-f (p12) | ATAGCATCTGGGGACAAAACGCCT |
| qFUB-r3 (p13) | AGCATCCGCACTACCCTTCTTAC |
| qFUB-f (p14) | TGATCTTCTTGCGAGTTGGGGTAT |
| B20_12133CheckF | CACGGCGCCTCATGTCCTTTGT |
| B20_12133CheckR | GATGAATTGGCCCCTCTTGGTGGTA |
