# Supplementary material for: Comparative genomics of geographically distant Fusarium fujikuroi isolates revealed two distinct pathotypes correlating with secondary metabolite profiles
Source: PLoS Pathog. 2017 Oct 26;13(10):e1006670. doi: 10.1371/journal.ppat.1006670 (PMC5675463; doi:10.1371/journal.ppat.1006670)
Supplement: S7 Table — (DOCX) [file ppat.1006670.s017.docx]

**Table S6:** **MRM transitions for liquid culture analysis in positive ionization mode, depicting also transition specific variables.**

| Q1 Mass (Da) | Q3 mass (Da) | Time (min) | ID | Window (sec) | Dwell weight | DP (V) | CE (V) |
| --- | --- | --- | --- | --- | --- | --- | --- |
| 321 | 261 | 8.21 | *O*-Me-FSR+H1 | 40 | 0.1 | 136 | 24 |
| 321 | 246 | 8.21 | *O*-Me-FSR+H2 | 40 | 0.1 | 136 | 33 |
| 321 | 233 | 8.21 | *O*-Me_FSR+H3 | 40 | 0.1 | 136 | 33 |
| 180 | 162 | 5.64 | FSA+H1 | 40 | 0.1 | 55 | 14 |
| 180 | 134 | 5.64 | FSA+H2 | 40 | 0.1 | 55 | 24 |
| 180 | 65 | 5.64 | FSA+H3 | 40 | 0.1 | 55 | 47 |
| 165 | 91 | 8.90 | GpyA+H1 | 40 | 1.0 | 130 | 30 |
| 165 | 77 | 8.90 | GpyA+H2 | 40 | 1.0 | 130 | 40 |
| 165 | 67 | 8.90 | GpyA+H3 | 40 | 1.0 | 130 | 23 |
| 663 | 84 | 10.65 | ApiF+H1 | 40 | 2.0 | 140 | 111 |
| 663 | 130 | 10.65 | ApiF+H2 | 40 | 2.0 | 140 | 130 |
| 663 | 112 | 10.65 | ApiF+H3 | 40 | 2.0 | 140 | 70 |
| 801 | 262 | 12.65 | BEA+NH_4_ 1 | 40 | 2.0 | 120 | 41 |
| 801 | 244 | 12.65 | BEA+NH_4_ 2 | 40 | 2.0 | 120 | 90 |
| 801 | 134 | 12.65 | BEA+NH_4_ 3 | 40 | 2.0 | 120 | 50 |
| 706 | 318 | 11.03 | FB2+H1 | 40 | 10.0 | 120 | 50 |
| 706 | 336 | 11.03 | FB2+H2 | 40 | 10.0 | 120 | 48 |
| 722 | 334 | 9.63 | FB1+H1 | 40 | 10.0 | 170 | 54 |
| 722 | 352 | 9.63 | FB1+H2 | 40 | 10.0 | 170 | 49 |
| 432 | 141 | 10.26 | FusC+H1 | 60 | 10.0 | 60 | 89 |
| 432 | 115 | 10.26 | FusC+H2 | 60 | 10.0 | 60 | 129 |
| 227 | 171 | 10.10 | Fujikurin+H1 | 60 | 5.0 | 180 | 15 |
| 227 | 191 | 10.10 | Fujikurin+H2 | 60 | 5.0 | 180 | 19 |
| 227 | 209 | 10.10 | Fujikurin+H3 | 60 | 5.0 | 180 | 15 |
