# Supplementary material for: Comparative genomics of geographically distant Fusarium fujikuroi isolates revealed two distinct pathotypes correlating with secondary metabolite profiles
Source: PLoS Pathog. 2017 Oct 26;13(10):e1006670. doi: 10.1371/journal.ppat.1006670 (PMC5675463; doi:10.1371/journal.ppat.1006670)
Supplement: S8 Table — (DOCX) [file ppat.1006670.s018.docx]

**Table S7: MRM transitions for liquid culture analysis in negative ionization mode, depicting also transition specific variables.**

| Q1 Mass (Da) | Q3 mass (Da) | Time (min) | ID | Window (sec) | Dwell weight | DP (V) | CE (V) |
| --- | --- | --- | --- | --- | --- | --- | --- |
| 151 | 92 | 6.54 | MePa-H1 | 40 | 1.0 | -210 | -30 |
| 151 | 136 | 6.54 | MePa-H2 | 40 | 1.0 | -210 | -19 |
| 345 | 283 | 6.36 | GA3-H1 | 40 | 10.0 | -165 | -20 |
| 345 | 227 | 6.36 | GA3-H2 | 40 | 10.0 | -165 | -35 |
| 345 | 143 | 6.36 | GA3-H3 | 40 | 10.0 | -165 | -40 |
| 331 | 243 | 9.64 | GA4-H1 | 40 | 10.0 | -180 | -25 |
| 331 | 257 | 9.64 | GA4-H2 | 40 | 10.0 | -180 | -33 |
| 331 | 213 | 9.64 | GA4-H3 | 40 | 10.0 | -180 | -40 |
| 329 | 285 | 9.46 | GA7-H1 | 40 | 10.0 | -165 | -24 |
| 329 | 223 | 9.46 | GA7-H2 | 40 | 10.0 | -165 | -25 |
| 329 | 211 | 9.46 | GA7-H3 | 40 | 10.0 | -165 | -36 |
